# Supplementary material for: A protease and a lipoprotein jointly modulate the conserved ExoR-ExoS-ChvI signaling pathway critical in Sinorhizobium meliloti for symbiosis with legume hosts
Source: PLoS Genet. 2023 Oct 23;19(10):e1010776. doi: 10.1371/journal.pgen.1010776 (PMC10659215; doi:10.1371/journal.pgen.1010776)
Supplement: S1 Fig — Ten-fold serial dilutions of logarithmic-phase cultures were spotted onto solid media and allowed to grow for three days prior to imaging. (A) Representative images show fluorescence of wild-type WSM419, ΔjspA (ΔSmed_3110) mutant, ΔlppA (ΔSmed_0632) mutant, and derivatives marked with neomycin (NmR) or spectinomycin (SpR) resistance [nptII or aadA linked to podJ (Smed_0147) or replacing jspA] on LB plates containing calcofluor. Darker spots indicate brighter fluorescence. (B) WSM419 and ΔjspA and ΔlppA mutants carrying the vector (pCM130) or a plasmid with S. meliloti jspA, jspAE148A, or lppA under the control of a taurine-inducible promoter (pJC535, pJC555, or pJC532, respectively) were grown on LB plates containing tetracycline (Tet) and calcofluor, without or with taurine (5 mM taurine for jspA complementation, 10 mM for lppA). Visible-light images of corresponding strains grown on PYE plates show mucoid colonies. Labels on the left indicate strain numbers, while labels on the right indicate genotypes. Each experiment was performed at least twice. (PDF) [file pgen.1010776.s001.pdf]

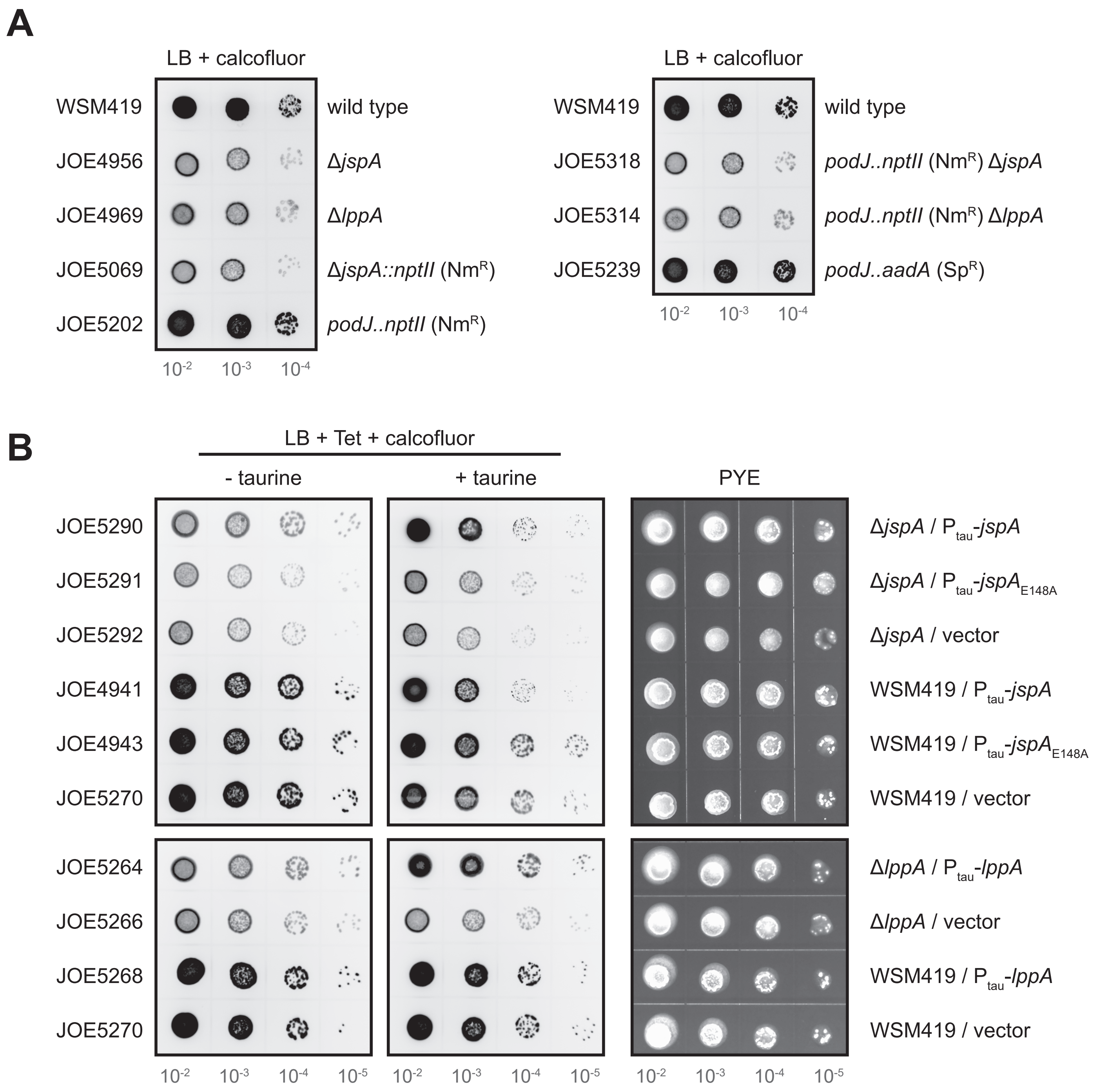

S1 Fig. Production of calcofluor-binding exopolysaccharides in *S. medicae* WSM419 and its derivatives. Ten-fold serial dilutions of logarithmic-phase cultures were spotted onto solid media and allowed to grow for three days prior to imaging. (**A**) Representative images show fluorescence of wild-type WSM419, Δ*jspA* (ΔSmed_3110) mutant, Δ*lppA* (ΔSmed_0632) mutant, and derivatives marked with neomycin (Nm^R) or spectinomycin (Sp^R) resistance [*nptII* or *aadA* linked to *podJ* (Smed_0147) or replacing *jspA*] on LB plates containing calcofluor. Darker spots indicate brighter fluorescence. (**B**) WSM419 and Δ*jspA* and Δ*lppA* mutants carrying the vector (pCM130) or a plasmid with *S. meliloti jspA*, $jspA_{E148A}$, or *lppA* under the control of a taurine-inducible promoter (pJC535, pJC555, or pJC532, respectively) were grown on LB plates containing tetracycline (Tet) and calcofluor, without or with taurine (5 mM taurine for *jspA* complementation, 10 mM for *lppA*). Visible-light images of corresponding strains grown on PYE plates show mucoid colonies. Labels on the left indicate strain numbers, while labels on the right indicate genotypes. Each experiment was performed at least twice.
