# Supplementary material for: A protease and a lipoprotein jointly modulate the conserved ExoR-ExoS-ChvI signaling pathway critical in Sinorhizobium meliloti for symbiosis with legume hosts
Source: PLoS Genet. 2023 Oct 23;19(10):e1010776. doi: 10.1371/journal.pgen.1010776 (PMC10659215; doi:10.1371/journal.pgen.1010776)
Supplement: S2 Fig — (A) Calcofluor fluorescence was used to assess EPS-I production in ΔlppA mutants expressing different alleles of lppA from a taurine-inducible promoter. (B) Residues altered in the LppA variants are depicted as ball-and-stick representations in the AlphaFold structural prediction (UniProt Q92R89) (which does not contain the additional 12 residues at the N-terminus that optimize the signal sequence). (C) Wild-type Rm1021 expressing different jspA alleles from a taurine-inducible promoter exhibit varying levels of fluorescence on calcofluor plates. (D) Immunoblot shows steady-state levels of different versions of JspA-HA. E148A-HA, E148D-HA, and H147A-HA stand for mutant versions of JspA-HA, encoded by jspAE148A-HA, jspAE148D-HA, and jspAH147A-HA, respectively. Samples were harvested from wild-type strains grown in LB with or without 10 mM taurine (+ or - taurine) for 3 hours. Numbers to the right of immunoblot indicate approximate molecular mass standards, in kDa. Plasmids pJC532, pJC605, pJC606, pJC607, pJC608, and pJC609 were used for expressing lppA, lppAC23S, lppA-HA, lppAC23S-HA, lppAG96W-HA, and lppAA78S-HA, while pJC535, pJC555, pJC556, pJC557, pJC558, pJC559, pJC560, pJC561 were used for jspA, jspAE148A, jspAE148D, jspAH147A, jspA-HA, jspAE148A-HA, jspAE148D-HA, and jspAH147A-HA, respectively. Vectors used were pCM130 (A, D) or pJC478 (C). For assessing calcofluor fluorescence, ten-fold serial dilutions (10−2 to 10−5) of logarithmic-phase cultures were spotted onto LB plates without or with taurine, and allowed to grow for three days prior to fluorescence imaging. Darker spots on representative images indicate brighter fluorescence. Portions of panels (A) and (C) are the same as images shown in Fig 6A and 6C. (PDF) [file pgen.1010776.s002.pdf]

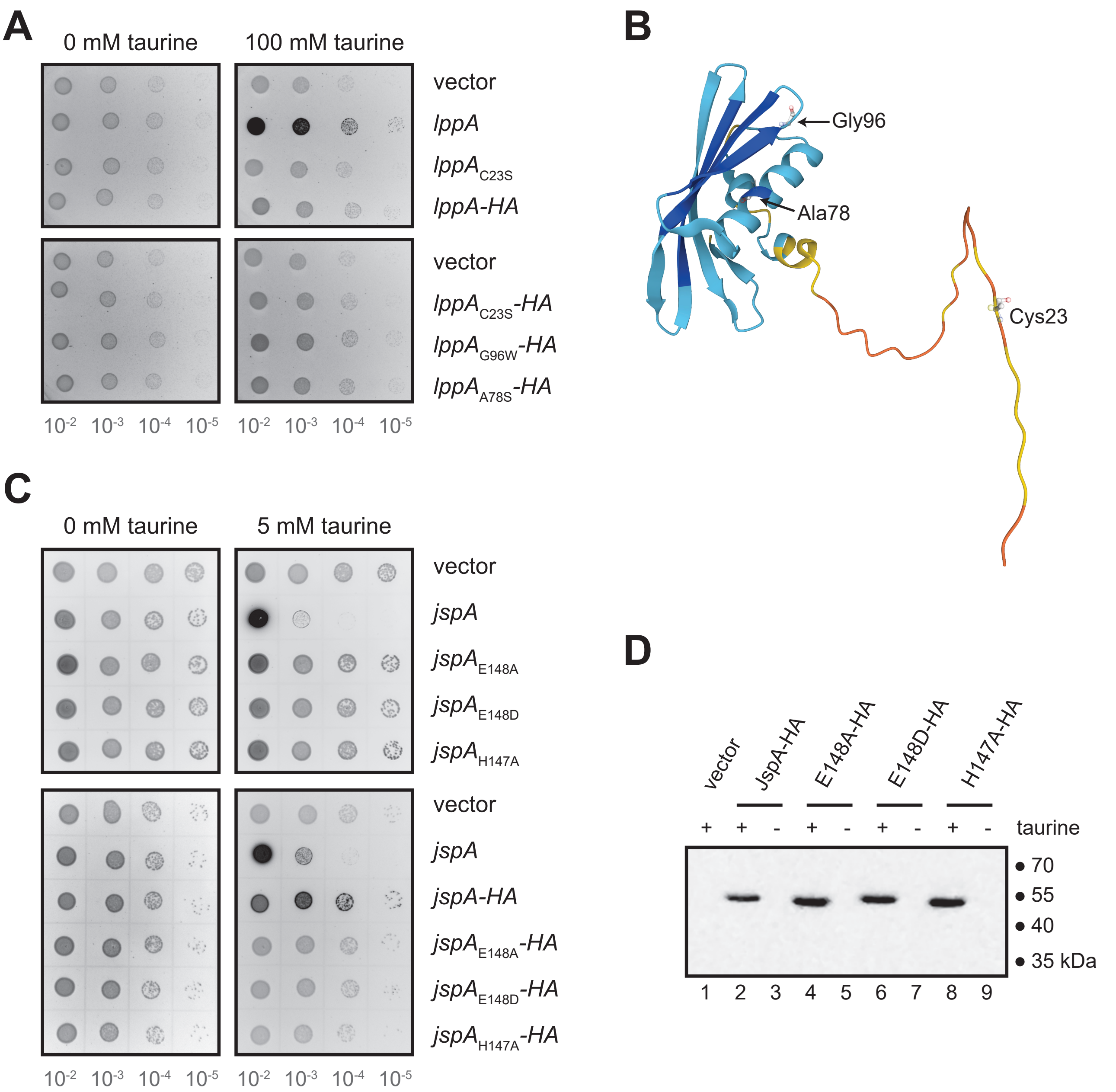

S2 Fig. Expression of mutant *lppA* and *jspA* alleles in *S. meliloti*. (**A**) Calcofluor fluorescence was used to assess EPS-I production in Δ*lppA* mutants expressing different alleles of *lppA* from a taurine-inducible promoter. (**B**) Residues altered in the LppA variants are depicted as ball-and-stick representations in the AlphaFold structural prediction (UniProt Q92R89) (which does not contain the additional 12 residues at the N-terminus that optimize the signal sequence). (**C**) Wild-type Rm1021 expressing different *jspA* alleles from a taurine-inducible promoter exhibit varying levels of fluorescence on calcofluor plates. (**D**) Immunoblot shows steady-state levels of different versions of JspA-HA. E148A-HA, E148D-HA, and H147A-HA stand for mutant versions of JspA-HA, encoded by *jspA*$_{E148A}$*-HA*, *jspA*$_{E148D}$*-HA*, and *jspA*$_{H147A}$*-HA*, respectively. Samples were harvested from wild-type strains grown in LB with or without 10 mM taurine (+ or - taurine) for 3 hours. Numbers to the right of immunoblot indicate approximate molecular mass standards, in kDa. Plasmids pJC532, pJC605, pJC606, pJC607, pJC608, and pJC609 were used for expressing *lppA*, *lppA*$_{C23S}$, *lppA-HA*, *lppA*$_{C23S}$*-HA*, *lppA*$_{G96W}$*-HA*, and *lppA*$_{A78S}$*-HA*, while pJC535, pJC555, pJC556, pJC557, pJC558, pJC559, pJC560, pJC561 were used for *jspA*, *jspA*$_{E148A}$, *jspA*$_{E148D}$, *jspA*$_{H147A}$, *jspA-HA*, *jspA*$_{E148A}$*-HA*, *jspA*$_{E148D}$*-HA*, and *jspA*$_{H147A}$*-HA*, respectively. Vectors used were pCM130 (A, D) or pJC478 (C). For assessing calcofluor fluorescence, ten-fold serial dilutions (10^{-2} to 10^{-5}) of logarithmic-phase cultures were spotted onto LB plates without or with taurine, and allowed to grow for three days prior to fluorescence imaging. Darker spots on representative images indicate brighter fluorescence. Portions of panels (A) and (C) are the same as images shown in Fig 6A and 6C.
