# Supplementary material for: A protease and a lipoprotein jointly modulate the conserved ExoR-ExoS-ChvI signaling pathway critical in Sinorhizobium meliloti for symbiosis with legume hosts
Source: PLoS Genet. 2023 Oct 23;19(10):e1010776. doi: 10.1371/journal.pgen.1010776 (PMC10659215; doi:10.1371/journal.pgen.1010776)
Supplement: S3 Fig — Ten-fold serial dilutions of logarithmic-phase cultures were spotted onto PYE plates containing 0, 5, or 10 mM taurine. C. crescentus NA1000 derivatives were grown with 1 μg/mL oxytetracycline for two days, while Sinorhizobium WSM419 and NGR234 derivatives were grown with 5 μg/mL oxytetracycline for three days at 30°C prior to imaging. Labels on the left indicate strain numbers, while labels on the right indicate the jspA alleles being expressed from a plasmid. Plasmids used were pJC614 (jspA), pJC615 (jspAE148A), pJC616 (jspA-HA), and pJC617 (jspAE148A-HA). Images shown represent four replicates on two different days. (PDF) [file pgen.1010776.s003.pdf]

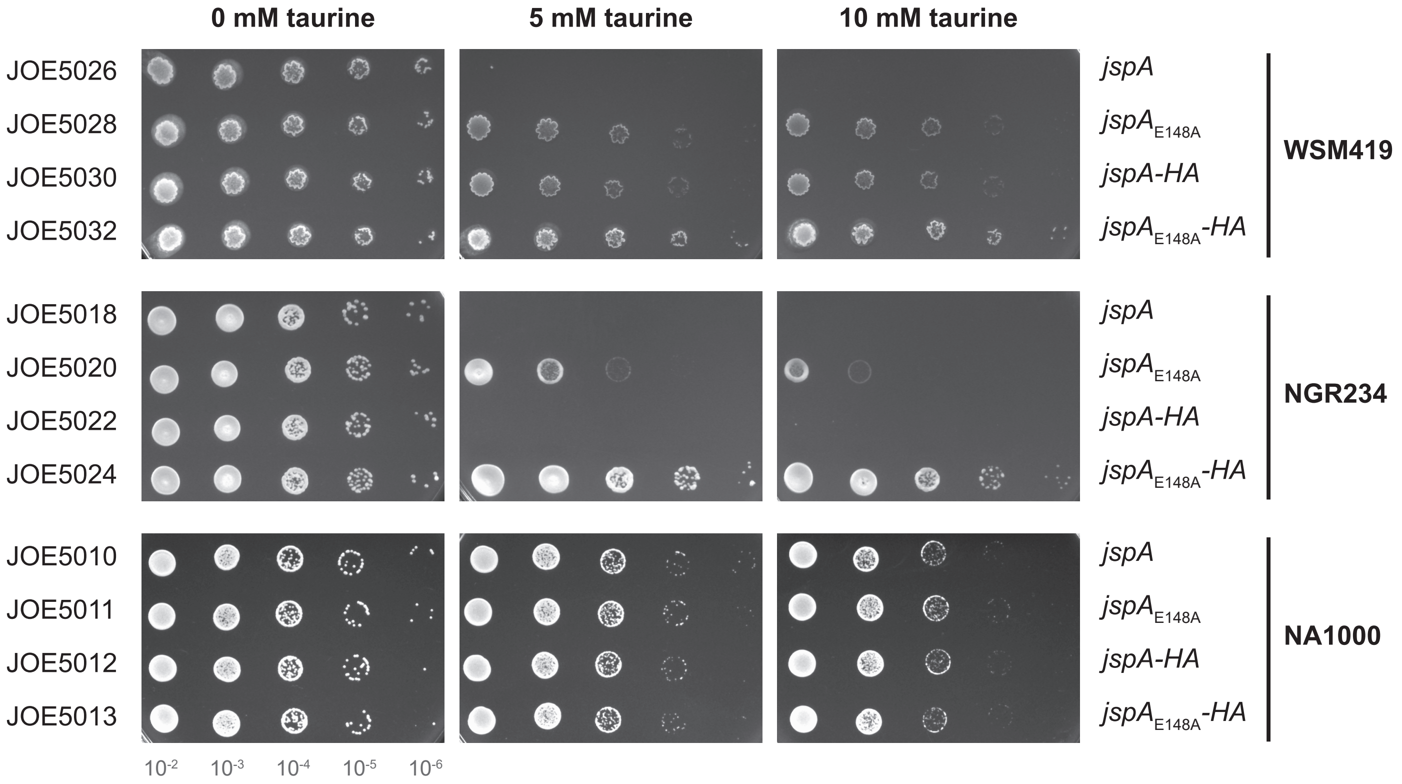

S3 Fig. Overexpression of *jspA* alleles in *S. medicae* WSM419, *S. fredii* NGR234, and *C. crescentus* NA1000. Ten-fold serial dilutions of logarithmic-phase cultures were spotted onto PYE plates containing 0, 5, or 10 mM taurine. *C. crescentus* NA1000 derivatives were grown with 1 µg/mL oxytetracycline for two days, while *Sinorhizobium* WSM419 and NGR234 derivatives were grown with 5 µg/mL oxytetracycline for three days at 30°C prior to imaging. Labels on the left indicate strain numbers, while labels on the right indicate the *jspA* alleles being expressed from a plasmid. Plasmids used were pJC614 (*jspA*), pJC615 ($jspA_{E148A}$), pJC616 (*jspA-HA*), and pJC617 ($jspA_{E148A}$-*HA*). Images shown represent four replicates on two different days.
