# Supplementary material for: A protease and a lipoprotein jointly modulate the conserved ExoR-ExoS-ChvI signaling pathway critical in Sinorhizobium meliloti for symbiosis with legume hosts
Source: PLoS Genet. 2023 Oct 23;19(10):e1010776. doi: 10.1371/journal.pgen.1010776 (PMC10659215; doi:10.1371/journal.pgen.1010776)
Supplement: S4 Fig — (A) Plate images show growth of ChvI depletion strain on LB medium in the presence and absence of 1 mM IPTG. The top strain (AD124) carried the pSRKKm vector and had a ΔchvI allelic replacement plasmid (pAD112) integrated into its chromosome but retained a copy of chvI+, while the bottom strain (AD115) carried pAD101, with chvI under the control of the Plac promoter, and had its chromosomal chvI replaced by a hygromycin resistance gene (hph). (B) Plots show growth curves of ChvI depletion strains over 24 hours in LB, in the presence or absence of 0.5 mM IPTG. Strains with chromosomal chvI+ or ΔchvI alleles carried pAD101, as well as a compatible vector (pCM130) or derivatives (pJC535 or pJC555) containing taurine-regulated jspA or jspAE184A (abbreviated as E148A), under control of the Ptau promoter. No taurine was added in these growth experiments. Cultures were shaken at 1000 rpm in 48-well plates, with 0.4 mL medium (containing kanamycin and oxytetracycline) per well. Absorbance at 600 nm (A600) was measured every 30 minutes. Average readings for three different days are shown, with corresponding color shadings indicating standard deviations. In the presence of IPTG, all strains exhibited similar growth patterns; curves for depletion strains carrying Ptau-jspA or jspAE148A grown with IPTG were omitted for clarity. Strains shown here for growth curves (JOE5579, JOE5604, JOE5606, JOE5608) all contain a genomic exoY-uidA reporter and constitute a subset of those used for GUS assays in Fig 8B. Absorbance readings and generation times calculated from growth curves are available in S9 Table. (PDF) [file pgen.1010776.s004.pdf]

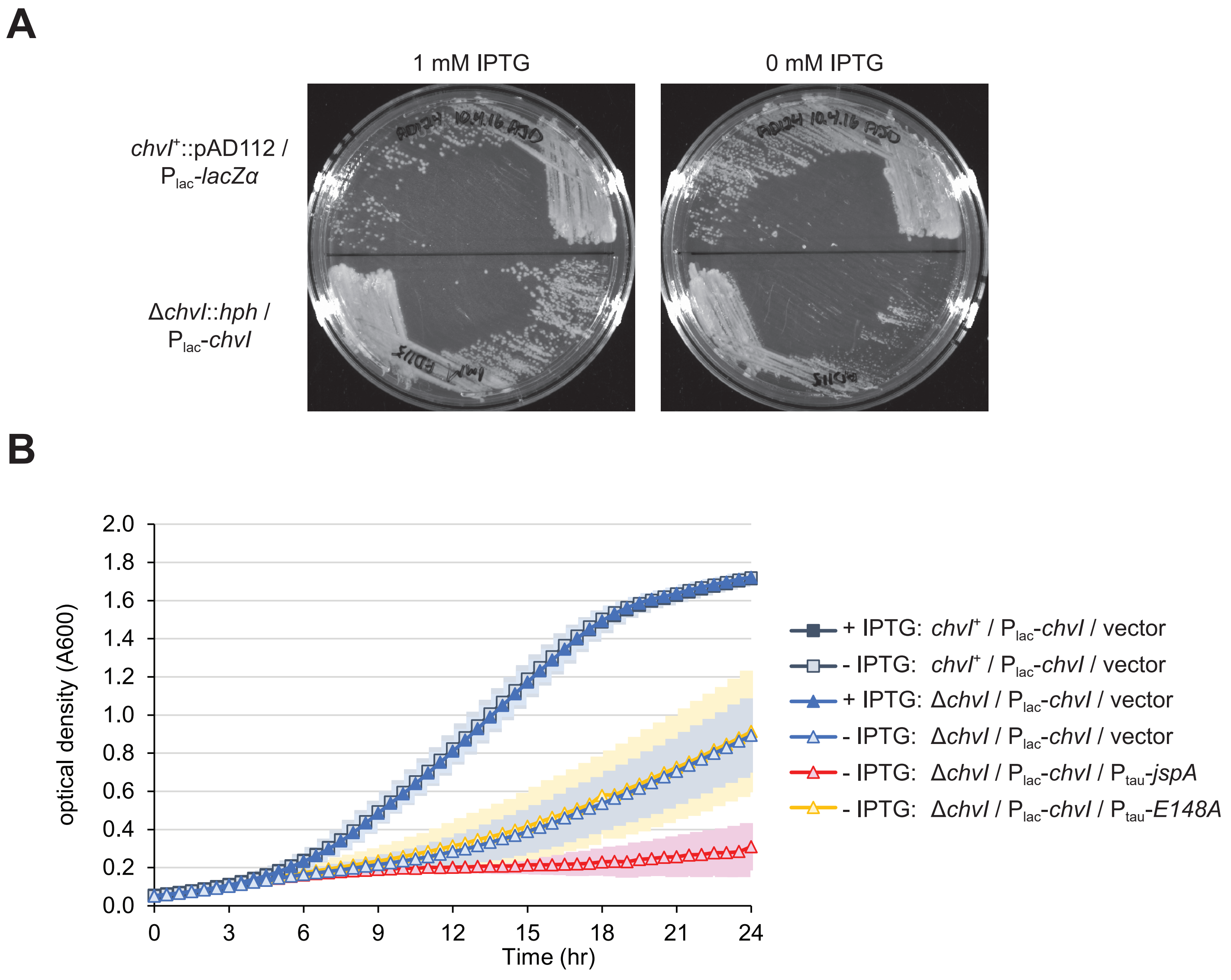

S4 Fig. Depletion of ChvI. (**A**) Plate images show growth of ChvI depletion strain on LB medium in the presence and absence of 1 mM IPTG. The top strain (AD124) carried the pSRKKm vector and had a Δ*chvI* allelic replacement plasmid (pAD112) integrated into its chromosome but retained a copy of *chvI*$^+$, while the bottom strain (AD115) carried pAD101, with *chvI* under the control of the P_{lac} promoter, and had its chromosomal *chvI* replaced by a hygromycin resistance gene (*hph*). (**B**) Plots show growth curves of ChvI depletion strains over 24 hours in LB, in the presence or absence of 0.5 mM IPTG. Strains with chromosomal *chvI*$^+$ or Δ*chvI* alleles carried pAD101, as well as a compatible vector (pCM130) or derivatives (pJC535 or pJC555) containing taurine-regulated *jspA* or *jspA*$_{E184A}$ (abbreviated as E148A), under control of the P_{tau} promoter. No taurine was added in these growth experiments. Cultures were shaken at 1000 rpm in 48-well plates, with 0.4 mL medium (containing kanamycin and oxytetracycline) per well. Absorbance at 600 nm (A600) was measured every 30 minutes. Average readings for three different days are shown, with corresponding color shadings indicating standard deviations. In the presence of IPTG, all strains exhibited similar growth patterns; curves for depletion strains carrying P_{tau}-*jspA* or *jspA*$_{E184A}$ grown with IPTG were omitted for clarity. Strains shown here for growth curves (JOE5579, JOE5604, JOE5606, JOE5608) all contain a genomic *exoY-uidA* reporter and constitute a subset of those used for GUS assays in Fig 8B. Absorbance readings and generation times calculated from growth curves are available in S9 Table.
