# Supplementary material for: A protease and a lipoprotein jointly modulate the conserved ExoR-ExoS-ChvI signaling pathway critical in Sinorhizobium meliloti for symbiosis with legume hosts
Source: PLoS Genet. 2023 Oct 23;19(10):e1010776. doi: 10.1371/journal.pgen.1010776 (PMC10659215; doi:10.1371/journal.pgen.1010776)
Supplement: S5 Fig — (A) Strains JOE5242 (carrying the pSRKGm vector), JOE5244 (with jspA on pJC652), and JOE5246 (with jspAE148A, noted as E148A, on pJC653) were grown in 48-well plates, with 0.4 mL PYE per well, in the presence of absence of 1 mM IPTG. Absorbance at 600 nm (A600) was measured every 30 minutes. Average readings for three different days are depicted, with surrounding shadings indicating standard deviations. Lines without markers represent growth in the absence of IPTG (-); standard deviations for these were omitted for simplicity. Fig 7 shows a portion of this graph. (B, C) Liquid cultures of the same strains were grown in flasks and induced with 1 mM IPTG in (B) PYE or (C) LB medium, and A600 was measured every hour for 12 hours. The plots for (B) and (C) were generated from single experiments. Absorbance readings and generation times calculated from growth curves are available in S9 Table. (PDF) [file pgen.1010776.s005.pdf]

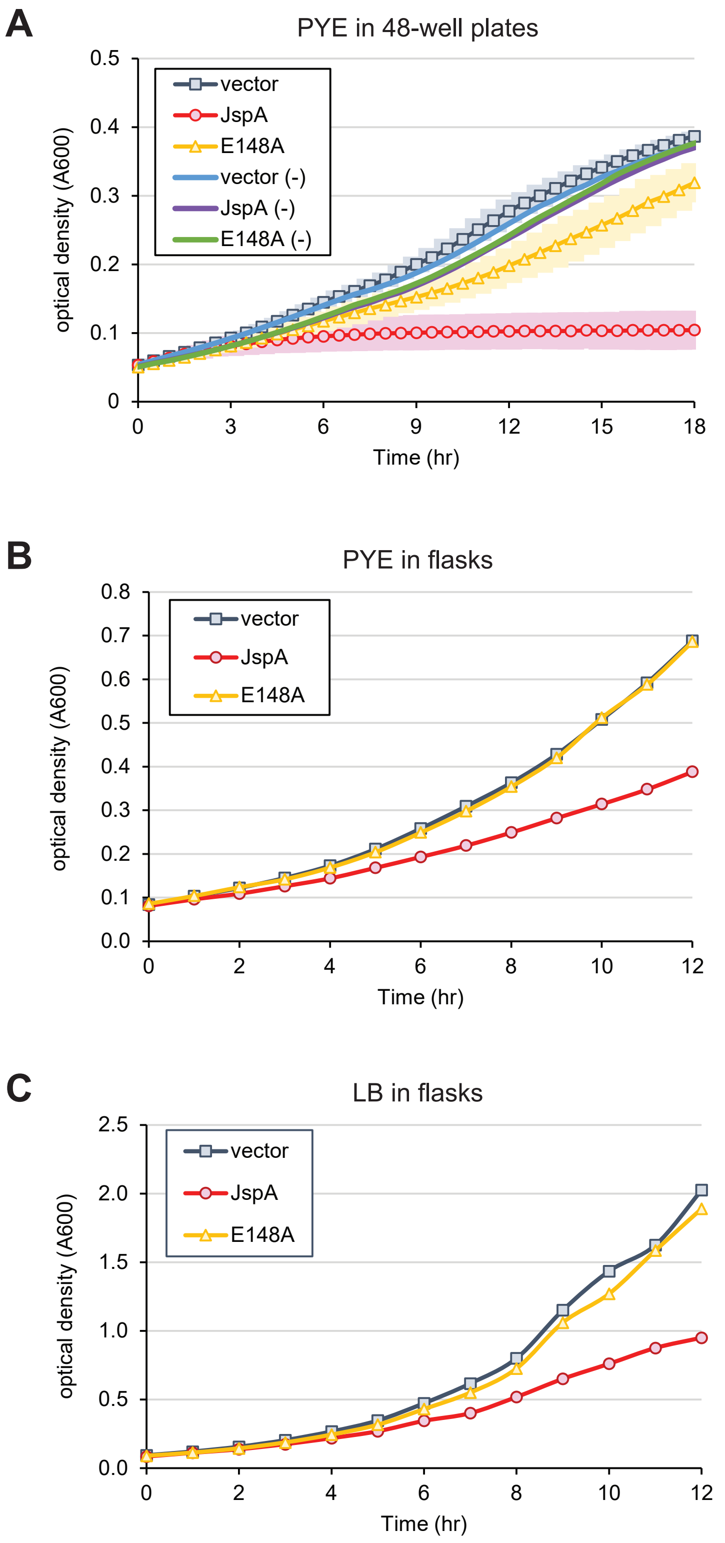

S5 Fig. Growth curves of *exoR-V5* strains expressing *jspA* alleles. (**A**) Strains JOE5242 (carrying the pSRKGm vector), JOE5244 (with *jspA* on pJC652), and JOE5246 (with $jspA_{E148A}$, noted as E148A, on pJC653) were grown in 48-well plates, with 0.4 mL PYE per well, in the presence of absence of 1 mM IPTG. Absorbance at 600 nm (A600) was measured every 30 minutes. Average readings for three different days are depicted, with surrounding shadings indicating standard deviations. Lines without markers represent growth in the absence of IPTG (-); standard deviations for these were omitted for simplicity. Fig 10 shows a portion of this graph. (**B, C**) Liquid cultures of the same strains were grown in flasks and induced with 1 mM IPTG in (B) PYE or (C) LB medium, and A600 was measured every hour for 12 hours. The plots for (B) and (C) were generated from single experiments. Absorbance readings and generation times calculated from growth curves are available in S9 Table.
