# Supplementary material for: A protease and a lipoprotein jointly modulate the conserved ExoR-ExoS-ChvI signaling pathway critical in Sinorhizobium meliloti for symbiosis with legume hosts
Source: PLoS Genet. 2023 Oct 23;19(10):e1010776. doi: 10.1371/journal.pgen.1010776 (PMC10659215; doi:10.1371/journal.pgen.1010776)
Supplement: S6 Fig — JspA, LppA, and ExoR-FLAG expression is indicated above the blot: - signifies no expression, + signifies expression of the wild-type allele, and *, #, and ^ represent JspAE148A, JspA-HA, and JspAE148A-HA, respectively. Positions of bands representing JspA-HA and ExoR-FLAG are indicated to the right of the blot; “pre” indicates the precursor form of ExoR-FLAG, “ExoR-FLAG” the mature form, and “deg” a major degradation product. Approximate molecular mass, in kDa, are shown to the left of the blots, while lane numbers are shown below. Expression of ExoR-FLAG from pMB859 was induced with 0.1 mM IPTG in PYE medium for 4 hours, while no expression means that the strain carried the empty vector pSRKKm under the same growth conditions. Expression of LppA and different versions of JspA were induced with 10 mM taurine from the following plasmids: lanes 1 and 4, empty vector pJC473 when neither expressed (- for both LppA and JspA); lanes 2 and 9, pJC616 for JspA-HA only (red #); lanes 3 and 10, pJC617 for JspAE148A-HA (red ^); lane 5, pJC614 for wild-type JspA (black +); lane 6, pJC615 for JspAE148A (black *); lane 7, pJC702 for LppA and JspA (black + for both); lane 8, pJC706 for LppA and JspAE148A (black + and *); lane 11, pJC707 for LppA and JspA-HA (black + and red #); and lane12, pJC708 for LppA and JspAE148A-HA (black + and red ^). The blot was first probed with anti-FLAG antibodies and then with anti-HA antibodies. This representative image was captured after both antibodies were applied. (PDF) [file pgen.1010776.s006.pdf]

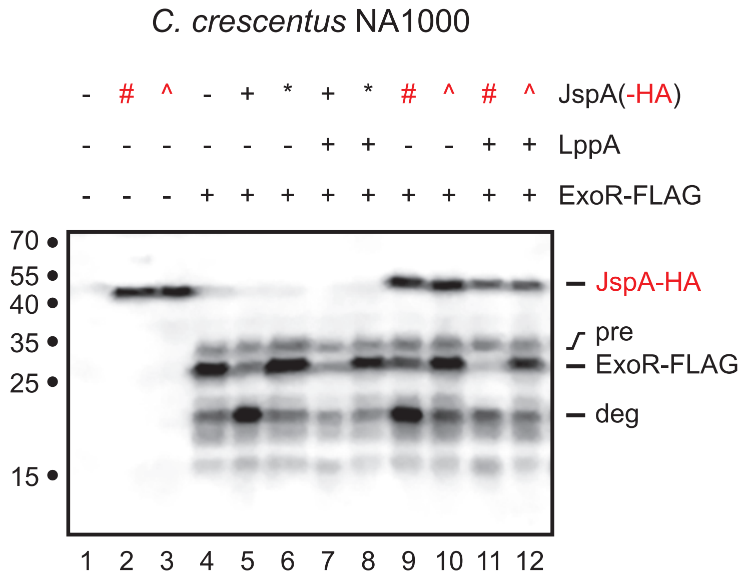

S6 Fig. Steady-state levels of ExoR-FLAG when co-expressed with LppA and different forms of JspA in *C. crescentus* NA1000. JspA, LppA, and ExoR-FLAG expression is indicated above the blot: - signifies no expression, + signifies expression of the wild-type allele, and *, #, and ^ represent $JspA_{E148A}$, JspA-HA, and $JspA_{E148A}$-HA, respectively. Positions of bands representing JspA-HA and ExoR-FLAG are indicated to the right of the blot; "pre" indicates the precursor form of ExoR-FLAG, "ExoR-FLAG" the mature form, and "deg" a major degradation product. Approximate molecular mass, in kDa, are shown to the left of the blots, while lane numbers are shown below. Expression of ExoR-FLAG from pMB859 was induced with 0.1 mM IPTG in PYE medium for 4 hours, while no expression means that the strain carried the empty vector pSRKKm under the same growth conditions. Expression of LppA and different versions of JspA were induced with 10 mM taurine from the following plasmids: lanes 1 and 4, empty vector pJC473 when neither expressed (- for both LppA and JspA); lanes 2 and 9, pJC616 for JspA-HA only (red #); lanes 3 and 10, pJC617 for $JspA_{E148A}$-HA (red ^); lane 5, pJC614 for wild-type JspA (black +); lane 6, pJC615 for $JspA_{E148A}$ (black *); lane 7, pJC702 for LppA and JspA (black + for both); lane 8, pJC706 for LppA and $JspA_{E148A}$ (black + and *); lane 11, pJC707 for LppA and JspA-HA (black + and red #); and lane12, pJC708 for LppA and $JspA_{E148A}$-HA (black + and red ^). The blot was first probed with anti-FLAG antibodies and then with anti-HA antibodies. This representative image was captured after both antibodies were applied.
