# Supplementary material for: A protease and a lipoprotein jointly modulate the conserved ExoR-ExoS-ChvI signaling pathway critical in Sinorhizobium meliloti for symbiosis with legume hosts
Source: PLoS Genet. 2023 Oct 23;19(10):e1010776. doi: 10.1371/journal.pgen.1010776 (PMC10659215; doi:10.1371/journal.pgen.1010776)
Supplement: S7 Fig — Symbols in rows above the blots indicate expression of different proteins, with - indicating no expression. For ExoR-FLAG, + signifies expression from pMB859, while - signifies carriage of the pSRKKm vector. For JspA-HA, red # and ^ indicate expression of wild-type JspA-HA and mutant JspAE148A-HA, respectively. For LppA-HA, the size of the + symbol reflects the level of expression, with the orange, bold + indicating the highest levels. Schematics above the blots represent gene arrangements on plasmids used for expressing JspA-HA variants and LppA-HA: red ^ indicates plasmids that carry the jspAE148A-HA mutant allele and the approximate location of the active site mutation in the gene; RBS preceding lppA-HA in pJC730 and pJC735 is the ribosome binding site of E. coli araB. Positions of bands representing JspA-HA, LppA-HA, and ExoR-FLAG are indicated to the right of the blots: “pre” indicates the precursor form of ExoR-FLAG, “ExoR-FLAG” the mature form, and “deg” a major degradation product; “read-through” indicates presumed fusions of JspA-HA and LppA-HA that resulted from translational read-through. LppA-HA expression is shown with two different blots because signals from strains with lower levels of expression (lanes 4, 5, 6, 7) were overwhelmed by those with the highest levels of expression (lanes 8, 9, 12, 13); the bottom image was captured using a duplicate blot without the highest-expression samples. Steady-state levels of pre-processed ExoR-FLAG appeared relatively high when expressed in E. coli DH10B compared to S. meliloti and C. crescentus, possibly due to inefficient export. Approximate molecular mass, in kDa, are shown to the left of the blots, while lane numbers are shown below. All strains were induced with 0.1 mM IPTG in LB medium for 4 hours. Plasmids used for expressing JspA-HA and LppA-HA are indicated in the schematics above the blots; lanes 1 and 14 used pDSW208 (expressing GFP) as the vector, lanes 10 and 11 used pJC720 and pJC733, while lanes 12 [file pgen.1010776.s007.pdf]

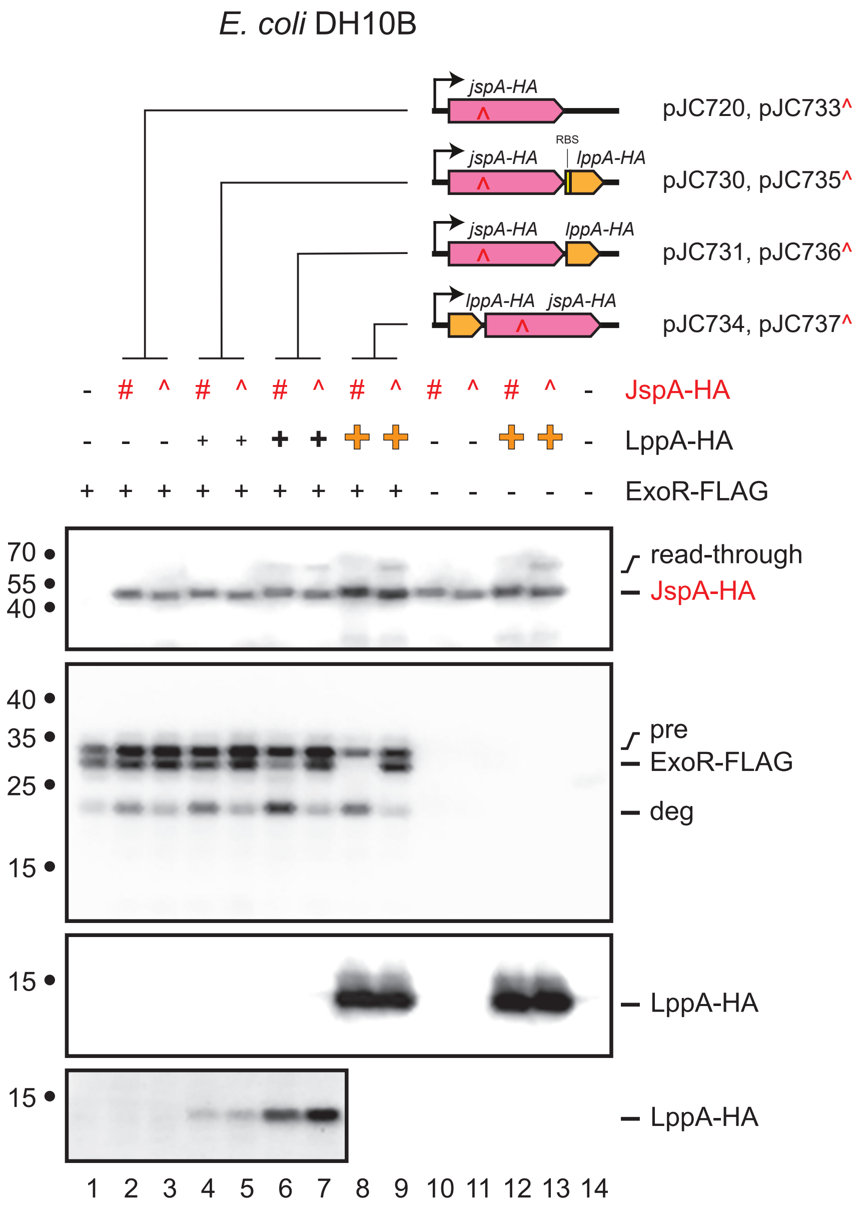

S7 Fig. Steady-state levels of ExoR-FLAG when co-expressed with wild-type or mutant JspA-HA and varying levels of LppA-HA in *E. coli* DH10B. Symbols in rows above the blots indicate expression of different proteins, with - indicating no expression. For ExoR-FLAG, + signifies expression from pMB859, while - signifies carriage of the pSRKKm vector. For JspA-HA, red # and ^ indicate expression of wild-type JspA-HA and mutant $\text{JspA}_{\text{E148A}}$-HA, respectively. For LppA-HA, the size of the + symbol reflects the level of expression, with the orange, bold + indicating the highest levels. Schematics above the blots represent gene arrangements on plasmids used for expressing JspA-HA variants and LppA-HA: red ^ indicates plasmids that carry the $\textit{jspA}_{\textit{E148A}}$*-HA* mutant allele and the approximate location of the active site mutation in the gene; RBS preceding *lppA-HA* in pJC730 and pJC735 is the ribosome binding site of *E. coli araB*. Positions of bands representing JspA-HA, LppA-HA, and ExoR-FLAG are indicated to the right of the blots: "pre" indicates the precursor form of ExoR-FLAG, "ExoR-FLAG" the mature form, and "deg" a major degradation product; "read-through" indicates presumed fusions of JspA-HA and LppA-HA that resulted from translational read-through. LppA-HA expression is shown with two different blots because signals from strains with lower levels of expression (lanes 4, 5, 6, 7) were overwhelmed by those with the highest levels of expression (lanes 8, 9, 12, 13); the bottom image was captured using a duplicate blot without the highest-expression samples. Steady-state levels of pre-processed ExoR-FLAG appeared relatively high when expressed in *E. coli* DH10B compared to *S. meliloti* and *C. crescentus*, possibly due to inefficient export. Approximate molecular mass, in kDa, are shown to the left of the blots, while lane numbers are shown below. All strains were induced with 0.1 mM IPTG in LB medium for 4 hours. Plasmids used for expressing JspA-HA and LppA-HA are indicated in the schematics above the blots; lanes 1 and 14 used pDSW208 (expressing GFP) as the vector, lanes 10 and 11 used pJC720 and pJC733, while lanes 12 and 13 used pJC734 and pJC737, respectively.
