# Supplementary material for: A protease and a lipoprotein jointly modulate the conserved ExoR-ExoS-ChvI signaling pathway critical in Sinorhizobium meliloti for symbiosis with legume hosts
Source: PLoS Genet. 2023 Oct 23;19(10):e1010776. doi: 10.1371/journal.pgen.1010776 (PMC10659215; doi:10.1371/journal.pgen.1010776)
Supplement: S11 Table — (DOCX) [file pgen.1010776.s018.docx]

**S11 Table.** *Sinorhizobium meliloti* and *medicae* strains used in this study

| **Strains** | **Relevant genetic markers, features, and/or description** | **Construction, source, or reference ^a^** |
| --- | --- | --- |
|  |  |  |
| Rm1021 | SU47 derivative, Sm^R^ (progenitor of strains listed below) | (Meade *et al.*, 1982) |
| Rm7095 | *exoR95*::Tn*5* | (Doherty *et al.*, 1988) |
| Rm7096 | *exoS96*::Tn*5* | (Doherty *et al.*, 1988) |
| CL150 | *ecfR1*^+^ *pstC*^+^ | (Schlüter *et al.*, 2013) |
| CSN015 | Δ*podJ1 lppA*::Tn*5*-110 (SMc00067::Tn*5*-110) | (Fields *et al.*, 2012) |
| CSN134 | Δ*podJ1 jspA*::Tn*5*-110 (SMc03872::Tn*5*-110) | (Fields *et al.*, 2012) |
| EC443 | *exoR-V5* | (Chen *et al.*, 2008) |
| MB669 | *P_flaC_*-*uidA* (SMc03040::pMB694) | (Gibson *et al.*, 2007) |
| AD084 | CL150 *P_exoY_*-*uidA* (*exoY*::pAD083) | pAD083 mated into CL150 |
| AD113 | CL150 *chvI*::pAD112 (pJQ200sk-Δ*chvI*::*hph*) | pAD112 mated into CL150 |
| AD114 | CL150 *chvI*::pAD112 / pAD101 (pSRKKm-*chvI*) | pAD101 mated into AD113 |
| AD115 | CL150 Δ*chvI*::*hph* / pAD101 (ChvI depletion strain) | Sucrose counter-selection of AD114 |
| AD124 | CL150 *chvI*::pAD112 / pSRKKm | pSRKKm mated into AD113 |
| JOE2612 | *podJ2*(SMc02231)..Ω (Sp^R^ wild-type strain, with antibiotic marker inserted after *podJ2*) | (Fields *et al.*, 2012) |
| JOE2968 | *P_flaC_*-*uidA* (SMc03040::pMB694) | (Fields *et al.*, 2012) |
| JOE3200 | Rm1021 / pCM130 | pCM130 mated into Rm1021 |
| JOE3249 | Δ*jspA* (ΔSMc03872) | (Fields *et al.*, 2012) |
| JOE3253 | *P_mcpU_*-*uidA* (SMc00975::pMB696) | pMB696 mated into Rm1021 (Fields *et al.*, 2012) |
| JOE3366 | Δ*lppA* (ΔSMc00067) | (Fields *et al.*, 2012) |
| JOE3597 | Rm1021 / pJC478 (pCM130-P_tau_-*uidA*) | pJC478 mated into Rm1021 |
| JOE3780 | Δ*lppA* *exoS96*::Tn*5* | JOE3366 x Φ(Rm7096), select for Nm^R^ |
| JOE3782 | Δ*jspA* *exoR95*::Tn*5* | JOE3249 x Φ(Rm7096), select for Nm^R^ |
| JOE3826 | Δ*lppA* *P_exoY_*-*uidA* (SMb20946::pEC340) | pEC340 mated into JOE3366 |
| JOE3827 | Δ*jspA* *P_exoY_*-*uidA* | pEC340 mated into JOE3249 |
| JOE3829 | *P_exoY_*-*uidA* | pEC340 mated into Rm1021 |
| JOE3902 | Δ*lppA* *exoR95*::Tn*5* | JOE3366 x Φ(Rm7095), select for Nm^R^ |
| JOE3903 | Δ*jspA* *exoR95*::Tn*5* | JOE3249 x Φ(Rm7095), select for Nm^R^ |
| JOE4041 | *lppA*::Tn*5*-110 | Rm1021 x Φ(CSN015), select for Nm^R^ |
| JOE4044 | *jspA*::Tn*5*-110 | Rm1021 x Φ(CSN134), select for Nm^R^ |
| JOE4134 | Δ*jspA* / pJC535 (pCM130-P_tau_-*jspA*) | pJC535 mated into JOE3249 |
| JOE4135 | Δ*jspA* / pCM130 | pCM130 mated into JOE3249 |
| JOE4136 | Δ*jspA* / pJC478 (pCM130-P_tau_-*uidA*) | pJC478 mated into JOE3249 |
| JOE4140 | Rm1021 / pJC535 | pJC535 mated into Rm1021 |
| JOE4141 | Δ*lppA* / pJC532 (pCM130-P_tau_-*lppA*) | pJC532 mated into JOE3366 |
| JOE4143 | Δ*lppA* / pCM130 | pCM130 mated into JOE3366 |
| JOE4149 | Rm1021 / pJC532 | pJC532 mated into Rm1021 |
| JOE4165 | Δ*jspA* / pJC532 | pJC532 mated into JOE3249 |
| JOE4189 | Δ*lppA* *P_flaC_*-*uidA* | JOE3366 x Φ(MB669), select for Sp^R^ |
| JOE4191 | Δ*jspA* *P_flaC_*-*uidA* | JOE3249 x Φ(MB669), select for Sp^R^ |
| JOE4206 | Δ*lppA* / pJC535 | pJC535 mated into JOE3366 |
| JOE4213 | *P_flaC_*-*uidA* / pCM130 | pCM130 mated into JOE2968 |
| JOE4215 | *P_flaC_*-*uidA* / pJC532 | pJC532 mated into JOE2968 |
| JOE4217 | *P_flaC_*-*uidA* / pJC535 | pJC535 mated into JOE2968 |
| JOE4219 | *P_exoY_*-*uidA* / pCM130 | pCM130 mated into JOE3829 |
| JOE4221 | *P_exoY_*-*uidA* / pJC532 | pJC532 mated into JOE3829 |
| JOE4223 | *P_exoY_*-*uidA* / pJC535 | pJC535 mated into JOE3829 |
| JOE4231 | *P_mcpU_*-*uidA* | Rm1021 x Φ(JOE3253), select for Sp^R^ |
| JOE4232 | Δ*jspA* *P_mcpU_*-*uidA* | JOE3249 x Φ(JOE3253), select for Sp^R^ |
| JOE4233 | Δ*lppA* *P_mcpU_*-*uidA* | JOE3366 x Φ(JOE3253), select for Sp^R^ |
| JOE4237 | *P_exoR_*-*uidA* (SMc02078::pJC540) | pJC540 mated into Rm1021 |
| JOE4273 | Δ*lppA* *P_exoY_*-*uidA* / pCM130 | pCM130 mated into JOE3826 |
| JOE4275 | Δ*lppA* *P_exoY_*-*uidA* / pJC532 | pJC532 mated into JOE3826 |
| JOE4277 | Δ*lppA* *P_exoY_*-*uidA* / pJC535 | pJC535 mated into JOE3826 |
| JOE4279 | Δ*jspA* *P_exoY_*-*uidA* / pCM130 | pCM130 mated into JOE3827 |
| JOE4281 | Δ*jspA* *P_exoY_*-*uidA* / pJC532 | pJC532 mated into JOE3827 |
| JOE4283 | Δ*jspA* *P_exoY_*-*uidA* / pJC535 | pJC535 mated into JOE3827 |
| JOE4285 | Δ*jspA* Δ*lppA* | Allelic replacement in JOE3249 using pJC454 |
| JOE4296 | Δ*jspA* Δ*lppA* *P_exoY_*-*uidA* | JOE4285 x Φ(JOE3829), select for Nm^R^ |
| JOE4299 | Δ*jspA* Δ*lppA* *P_mcpU_*-*uidA* | JOE4285 x Φ(JOE3253), select for Sp^R^ |
| JOE4302 | Δ*jspA* Δ*lppA* *P_flaC_*-*uidA* | JOE4285 x Φ(MB669), select for Sp^R^ |
| JOE4400 | Rm1021 / pJC555 (pCM130-P_tau_-*jspA*_E148A_) | pJC555 mated into Rm1021 |
| JOE4402 | Rm1021 / pJC556 (pCM130-P_tau_-*jspA*_E148D_) | pJC556 mated into Rm1021 |
| JOE4404 | Rm1021 / pJC557 (pCM130-P_tau_-*jspA*_H147A_) | pJC557 mated into Rm1021 |
| JOE4406 | Rm1021 / pJC558 (pCM130-P_tau_-*jspA-HA*) | pJC558 mated into Rm1021 |
| JOE4408 | Rm1021 / pJC559 (pCM130-P_tau_-*jspA*_E148A_) | pJC559 mated into Rm1021 |
| JOE4410 | Rm1021 / pJC560 (pCM130-P_tau_-*jspA*_E148D_) | pJC560 mated into Rm1021 |
| JOE4412 | Rm1021 / pJC561 (pCM130-P_tau_-*jspA*_H147A_) | pJC561 mated into Rm1021 |
| JOE4521 | *P_exoY_*-*uidA* / pJC555 | pJC555 mated into JOE3829 |
| JOE4524 | *P_exoY_*-*uidA* / pJC556 | pJC556 mated into JOE3829 |
| JOE4527 | *P_exoY_*-*uidA* / pJC558 | pJC558 mated into JOE3829 |
| JOE4529 | *P_exoY_*-*uidA* / pJC559 | pJC559 mated into JOE3829 |
| JOE4531 | *P_exoY_*-*uidA* / pJC560 | pJC560 mated into JOE3829 |
| JOE4723 | *P_mcpU_*-*uidA* / pCM130 | pCM130 mated into JOE3253 |
| JOE4725 | *P_mcpU_*-*uidA* / pJC535 | pJC535 mated into JOE3253 |
| JOE4727 | *P_mcpU_*-*uidA* / pJC555 | pJC555 mated into JOE3253 |
| JOE4729 | *P_mcpU_*-*uidA* / pJC556 | pJC556 mated into JOE3253 |
| JOE4731 | *P_exoR_*-*uidA* / pCM130 | pCM130 mated into JOE4237 |
| JOE4733 | *P_exoR_*-*uidA* / pJC535 | pJC535 mated into JOE4237 |
| JOE4735 | *P_exoR_*-*uidA* / pJC555 | pJC555 mated into JOE4237 |
| JOE4737 | *P_exoR_*-*uidA* / pJC556 | pJC556 mated into JOE4237 |
| JOE4760 | Δ*lppA* *P_exoY_*-*uidA* / pJC558 | pJC558 mated into JOE3826 |
| JOE4762 | Δ*jspA* *P_exoY_*-*uidA* / pJC558 | pJC558 mated into JOE3827 |
| JOE4764 | Δ*lppA* *P_exoY_*-*uidA* / pJC559 | pJC559 mated into JOE3826 |
| JOE4766 | Δ*jspA* *P_exoY_*-*uidA* / pJC559 | pJC559 mated into JOE3827 |
| JOE4873 | Δ*lppA* *P_exoY_*-*uidA* / pJC605 (P_tau_-*lppA*_C23S_) | pJC605 mated into JOE3826 |
| JOE4874 | Δ*lppA* *P_exoY_*-*uidA* / pJC606 (P_tau_-*lppA-HA*) | pJC606 mated into JOE3826 |
| JOE4875 | Δ*lppA* *P_exoY_*-*uidA* / pJC607 (P_tau_-*lppA*_C23S_*-HA*) | pJC607 mated into JOE3826 |
| JOE4876 | Δ*lppA* *P_exoY_*-*uidA* / pJC608 (P_tau_-*lppA*_G96W_*-HA*) | pJC608 mated into JOE3826 |
| JOE4877 | Δ*lppA* *P_exoY_*-*uidA* / pJC609 (P_tau_-*lppA*_A78S_*-HA*) | pJC609 mated into JOE3826 |
| JOE5154 | *P_chvI_*-*uidA* (SMc02560::pJC638) | pJC638 mated into Rm1021 |
| JOE5155 | *P_pckA_*-*uidA* (SMc02562::pJC639) | pJC639 mated into Rm1021 |
| JOE5156 | *P*_SMc01580_-*uidA* (SMc01580::pJC640) | pJC640 mated into Rm1021 |
| JOE5157 | Δ*jspA* *P_chvI_*-*uidA* | pJC638 mated into JOE3249 |
| JOE5159 | Δ*jspA P*_SMc01580_-*uidA* | pJC640 mated into JOE3249 |
| JOE5160 | Δ*lppA* *P_chvI_*-*uidA* | pJC638 mated into JOE3366 |
| JOE5162 | Δ*lppA P*_SMc01580_-*uidA* | pJC640 mated into JOE3366 |
| JOE5209 | *P_chvI_*-*uidA* / pCM130 | pCM130 mated into JOE5154 |
| JOE5210 | *P_chvI_*-*uidA* / pJC535 | pJC535 mated into JOE5154 |
| JOE5211 | *P_chvI_*-*uidA* / pJC555 | pJC555 mated into JOE5154 |
| JOE5212 | *P*_SMc01580_-*uidA* / pCM130 | pCM130 mated into JOE5156 |
| JOE5213 | *P*_SMc01580_-*uidA* / pJC535 | pJC535 mated into JOE5156 |
| JOE5214 | *P*_SMc01580_-*uidA* / pJC555 | pJC555 mated into JOE5156 |
| JOE5219 | *P_chvI_*-*uidA* / pJC652 (pSRKGm-*jspA*) | pJC652 mated into JOE5154 |
| JOE5220 | *P_chvI_*-*uidA* / pJC653 (pSRKGm-*jspA*_E148A_) | pJC653 mated into JOE5154 |
| JOE5221 | *P_chvI_*-*uidA* / pJC654 (pSRKGm-*jspA-HA*) | pJC654 mated into JOE5154 |
| JOE5222 | *P_chvI_*-*uidA* / pJC655 (pSRKGm-*jspA*_E148A_*-HA*) | pJC655 mated into JOE5154 |
| JOE5242 | *exoR-V5* / pSRKGm | pSRKGm mated into EC443 |
| JOE5244 | *exoR-V5* / pJC652 | pJC652 mated into EC443 |
| JOE5246 | *exoR-V5* / pJC653 | pJC653 mated into EC443 |
| JOE5252 | *P_chvI_*-*uidA* / pSRKGm | pSRKGm mated into JOE5154 |
| JOE5254 | *P_pckA_*-*uidA* / pCM130 | pCM130 mated into JOE5155 |
| JOE5256 | *P_pckA_*-*uidA* / pJC535 | pJC535 mated into JOE5155 |
| JOE5258 | *P_pckA_*-*uidA* / pJC555 | pJC555 mated into JOE5155 |
| JOE5354 | *P*_SMb21188_-*uidA* (SMb21188::pEC571) | pEC571 mated into Rm1021 |
| JOE5360 | Δ*jspA* *P*_SMb21188_-*uidA* | pEC571 mated into JOE3249 |
| JOE5366 | Δ*lppA* *P*_SMb21188_-*uidA* | pEC571 mated into JOE3366 |
| JOE5450 | *P_exoY_*-*uidA* (*exoY*::pAD083) | Rm1021 x Φ(AD084), select for Sp^R^ |
| JOE5526 | *P_exoY_*-*uidA* / pAD101 (pSRKKm-*chvI*) | pAD101 mated into JOE5450 |
| JOE5528 | *P_exoY_*-*uidA* / pSRKKm | pSRKKm mated into JOE5450 |
| JOE5579 | *P_exoY_*-*uidA* / pAD101 / pCM130 | pCM130 mated into JOE5526 |
| JOE5580 | *P_exoY_*-*uidA* / pAD101 / pJC535 | pJC535 mated into JOE5526 |
| JOE5581 | *P_exoY_*-*uidA* / pAD101 / pJC555 | pJC555 mated into JOE5526 |
| JOE5585 | *P_exoY_*-*uidA* / pSRKKm / pCM130 | pCM130 mated into JOE5528 |
| JOE5586 | *P_exoY_*-*uidA* / pSRKKm / pJC535 | pJC535 mated into JOE5528 |
| JOE5604 | Δ*chvI*::*hph P_exoY_*-*uidA* / pAD101 / pCM130 | JOE5579 x Φ(AD115), select for Hy^R^ |
| JOE5606 | Δ*chvI*::*hph P_exoY_*-*uidA* / pAD101 / pJC535 | JOE5580 x Φ(AD115), select for Hy^R^ |
| JOE5608 | Δ*chvI*::*hph P_exoY_*-*uidA* / pAD101 / pJC555 | JOE5581 x Φ(AD115), select for Hy^R^ |
|  |  |  |
| WSM419 | *S. medicae* isolate from *M. murex* root nodule collected in Sardinia, Italy (progenitor of strains listed below) | (Reeve *et al.*, 2010) |
| JOE4941 | WSM419 / pJC535 (pCM130-P_tau_-*jspA*) | pJC535 mated into WSM419 |
| JOE4943 | WSM419 / pJC555 (pCM130-P_tau_-*jspA*_E148A_) | pJC555 mated into WSM419 |
| JOE4956 | WSM419 Δ*jspA* (ΔSmed_3110) | Allelic replacement using pJC611 |
| JOE4969, 4970 | WSM419 Δ*lppA* (ΔSmed_0632) | Allelic replacement using pJC610 |
| JOE5069 | WSM419 Δ*jspA* (ΔSmed_3110)::*nptII* | Allelic replacement using pJC622 |
| JOE5202 | WSM419 *podJ*(Smed_0147)..*nptII* (Nm^R^ wild-type strain, with antibiotic marker inserted after *podJ*) | Allelic replacement using pJC645 |
| JOE5239 | WSM419 *podJ*(Smed_0147)..*aadA* (Sp^R^ wild-type strain, with antibiotic marker inserted after *podJ*) | Allelic replacement using pJC642 |
| JOE5264 | WSM419 Δ*lppA* / pJC532 (pCM130-P_tau_-*lppA*) | pJC532 mated into JOE4970 |
| JOE5266 | WSM419 Δ*lppA* / pCM130 | pCM130 mated into JOE4970 |
| JOE5268 | WSM419 / pJC532 | pJC532 mated into WSM419 |
| JOE5270 | WSM419 / pCM130 | pCM130 mated into WSM419 |
| JOE5290 | WSM419 Δ*jspA* / pJC535 | pJC535 mated into JOE4956 |
| JOE5291 | WSM419 Δ*jspA* / pJC555 | pJC555 mated into JOE4956 |
| JOE5292 | WSM419 Δ*jspA* / pCM130 | pCM130 mated into JOE4956 |
| JOE5314 | WSM419 *podJ*..*nptII* Δ*lppA* | Allelic replacement in JOE4970 using pJC645 |
| JOE5318 | WSM419 *podJ*..*nptII* Δ*jspA* | Allelic replacement in JOE4956 using pJC645 |
|  |  |  |

^a^ Φ indicates generalized transduction, as mediated by bacteriophage ΦN3. For example, Rm1021 x Φ(CSN015) means that a bacteriophage lysate made from CSN015 was used to infect Rm1021. ChvI depletion strains were constructed in the presence of 0.5 - 1 mM IPTG.

**References**

Chen, E.J., Sabio, E.A., and Long, S.R. (2008) The periplasmic regulator ExoR inhibits ExoS/ChvI two-component signalling in *Sinorhizobium meliloti*. *Mol Microbiol* **69**: 1290-1303.

Doherty, D., Leigh, J.A., Glazebrook, J., and Walker, G.C. (1988) Rhizobium meliloti mutants that overproduce the *R. meliloti* acidic calcofluor-binding exopolysaccharide. *J Bacteriol* **170**: 4249-4256.

Fields, A.T., Navarrete, C.S., Zare, A.Z., Huang, Z., Mostafavi, M., Lewis, J.C., Rezaeihaghighi, Y., Brezler, B.J., Ray, S., Rizzacasa, A.L., Barnett, M.J., Long, S.R., Chen, E.J., and Chen, J.C. (2012) The conserved polarity factor *podJ1* impacts multiple cell envelope-associated functions in *Sinorhizobium meliloti*. *Mol Microbiol* **84**: 892-920.

Gibson, K.E., Barnett, M.J., Toman, C.J., Long, S.R., and Walker, G.C. (2007) The symbiosis regulator CbrA modulates a complex regulatory network affecting the flagellar apparatus and cell envelope proteins. *J Bacteriol* **189**: 3591-3602.

Meade, H.M., Long, S.R., Ruvkun, G.B., Brown, S.E., and Ausubel, F.M. (1982) Physical and genetic characterization of symbiotic and auxotrophic mutants of *Rhizobium meliloti* induced by transposon Tn5 mutagenesis. *J Bacteriol* **149**: 114-122.

Reeve, W., Chain, P., O'Hara, G., Ardley, J., Nandesena, K., Brau, L., Tiwari, R., Malfatti, S., Kiss, H., Lapidus, A., Copeland, A., Nolan, M., Land, M., Hauser, L., Chang, Y.J., Ivanova, N., Mavromatis, K., Markowitz, V., Kyrpides, N., Gollagher, M., Yates, R., Dilworth, M., and Howieson, J. (2010) Complete genome sequence of the *Medicago* microsymbiont *Ensifer* (*Sinorhizobium*) *medicae* strain WSM419. *Stand Genomic Sci* **2**: 77-86.

Schlüter, J.P., Reinkensmeier, J., Barnett, M.J., Lang, C., Krol, E., Giegerich, R., Long, S.R., and Becker, A. (2013) Global mapping of transcription start sites and promoter motifs in the symbiotic alpha-proteobacterium *Sinorhizobium meliloti* 1021. *BMC Genomics* **14**: 156.
