# Supplementary material for: A protease and a lipoprotein jointly modulate the conserved ExoR-ExoS-ChvI signaling pathway critical in Sinorhizobium meliloti for symbiosis with legume hosts
Source: PLoS Genet. 2023 Oct 23;19(10):e1010776. doi: 10.1371/journal.pgen.1010776 (PMC10659215; doi:10.1371/journal.pgen.1010776)
Supplement: S12 Table — (DOCX) [file pgen.1010776.s019.docx]

**S12 Table.** Plasmids used in this study

| **Plasmid** | **Relevant genetic markers, features, and/or description** | **Construction, source, or reference ^a^** |
| --- | --- | --- |
|  |  |  |
| pBAD18 | Arabinose-regulated expression, pBBR322 *ori*, Ap^R^ | (Guzman *et al.*, 1995) |
| pBbB2k-GFP | pBBR1-based broad-host-range plasmid, *gfp* under control of TetR-regulated promoter | (Lee *et al.*, 2011) |
| pCM130 | RK2-derived broad-host-range vector with *E. coli* *rrnB* terminator preceding polylinker, Tc^R^ | (Marx & Lidstrom, 2001) |
| pDSW204 | pTrc99A with weakened P_trc_, *lacI*^q^, Ap^R^, pBR322 *ori* | (Weiss *et al.*, 1999) |
| pDSW208 | pDSW204-*gfp* | (Weiss *et al.*, 1999) |
| pHP45Ω | Source of Ω cassette and *aadA* that confers Sm^R^/Sp^R^ | (Blondelet-Rouault *et al.*, 1997) |
| pJQ200sk | Counter-selectable vector for allelic replacement, *sacB*, Gm^R^ | (Quandt & Hynes, 1993) |
| pSRKGm | pBBR1MCS-derived broad-host-range vector, *lacI*-P_lac_, Gm^R^ | (Khan *et al.*, 2008) |
| pSRKKm | pBBR1MCS-derived broad-host-range vector, *lacI*-P_lac_, Nm^R^/Km^R^ | (Khan *et al.*, 2008) |
| pVO155 | pUC119-derived suicide vector with *uidA*^+^ cassette, Nm^R^/Km^R^ Ap^R^ | (Oke & Long, 1999) |
| pVO205 | pBluescript-derived plasmid, source of *hph* (Hy^R^) cassette | (Barnett *et al.*, 2000) |
| pVO345 | pUC119-derived suicide vector with *uidA*^+^ cassette, Sp^R^ Ap^R^ | (Gibson *et al.*, 2007) |
| pEC340 | pVO155-P*_exoY_* | (Chen *et al.*, 2009) |
| pEC571 | pVO155-P_SMb21188_ | (Chen *et al.*, 2009) |
| pMB694 | pVO345-P*_flaC_* | (Gibson *et al.*, 2007) |
| pMB696 | pVO345 P*_mcpU_* | (Gibson *et al.*, 2007) |
| pMB859 | pSRKKm-*exoR-FLAG* | This study |
| pAD083 | pVO345-P*_exoY_* | This study |
| pAD101 | pSRKKm-*chvI* | This study |
| pAD111 | pJQ200sk-Δ*chvI* | This study |
| pAD112 | pJQ200sk-Δ*chvI*::*hph* | This study |
| pJC382 | pJQ200sk-SMc02231..Ω (for inserting Sp^R^ marker between SMc02231 and SMc02232) | (Fields *et al.*, 2012) |
| pJC440 | pJQ200sk-Δ*jspA* (ΔSMc03872) | (Fields *et al.*, 2012) |
| pJC454 | pJQ200sk-Δ*lppA* (ΔSMc00067) | (Fields *et al.*, 2012) |
| pJC472 | pCM130-P_tau_ | (Mostafavi *et al.*, 2014) |
| pJC473 | pCM130-*tauR*-P_tau_ | (Mostafavi *et al.*, 2014) |
| pJC478 | pCM130-P_tau_-*uidA* | (Mostafavi *et al.*, 2014) |
| pJC532 | pCM130-P_tau_-*lppA* | This study |
| pJC535 | pCM130-P_tau_-*jspA* | This study |
| pJC540 | pVO155-P*_exoR_* | This study |
| pJC555 | pCM130-P_tau_-*jspA*_E148A_ | Megaprimer mutagenesis |
| pJC556 | pCM130-P_tau_-*jspA*_E148D_ | Megaprimer mutagenesis |
| pJC557 | pCM130-P_tau_-*jspA*_H147A_ | Megaprimer mutagenesis |
| pJC558 | pCM130-P_tau_-*jspA-HA* | This study |
| pJC559 | pCM130-P_tau_-*jspA*_E148A_*-HA* | This study |
| pJC560 | pCM130-P_tau_-*jspA*_E148D_*-HA* | This study |
| pJC561 | pCM130-P_tau_-*jspA*_H147A_*-HA* | This study |
| pJC605 | pCM130-P_tau_-*lppA*_C23S_ | Megaprimer mutagenesis |
| pJC606 | pCM130-P_tau_-*lppA-HA* | This study |
| pJC607 | pCM130-P_tau_-*lppA*_C23S_*-HA* | This study |
| pJC608 | pCM130-P_tau_-*lppA*_G96W_*-HA* | PCR error |
| pJC609 | pCM130-P_tau_-*lppA*_A78S_*-HA* | PCR error |
| pJC610 | pJQ200sk-Δ*lppA*_WSM419_ (ΔSmed_0632) | This study |
| pJC611 | pJQ200sk-Δ*jspA*_WSM419_ (ΔSmed_3110) | This study |
| pJC613 | pJQ200sk-Smed_0147 (for inserting markers after *podJ*_WSM419_) | This study |
| pJC614 | pCM130-*tauR*-P_tau_-*jspA* | This study |
| pJC615 | pCM130-*tauR*-P_tau_-*jspA*_E148A_ | This study |
| pJC616 | pCM130-*tauR*-P_tau_-*jspA-HA* | This study |
| pJC617 | pCM130-*tauR*-P_tau_-*jspA*_E148A_*-HA* | This study |
| pJC622 | pJQ200sk-Δ*jspA*_WSM419_::*nptII* | This study |
| pJC638 | pVO155-P*_chvI_* | This study |
| pJC639 | pVO155-P*_pckA_* | This study |
| pJC640 | pVO155-P_SMc01580_ | This study |
| pJC642 | pJQ200sk-Smed_0147..*aadA* (for inserting Sp^R^ marker after *podJ*) | This study |
| pJC645 | pJQ200sk-Smed_0147..*nptII* (for inserting Nm^R^ marker after *podJ*) | This study |
| pJC652 | pSRKGm-*jspA* | This study |
| pJC653 | pSRKGm-*jspA*_E148A_ | This study |
| pJC654 | pSRKGm-*jspA-HA* | This study |
| pJC655 | pSRKGm-*jspA*_E148A_*-HA* | This study |
| pJC700 | pCM130-*tauR*-P_tau_-*lppA* | This study |
| pJC702 | pCM130-*tauR*-P_tau_-*lppA-jspA* | This study |
| pJC706 | pCM130-*tauR*-P_tau_-*lppA-jspA*_E148A_ | This study |
| pJC707 | pCM130-*tauR*-P_tau_-*lppA-jspA-HA* | This study |
| pJC708 | pCM130-*tauR*-P_tau_-*lppA-jspA*_E148A_*-HA* | This study |
| pJC715 | pBAD18-*jspA-HA* | This study |
| pJC716 | pBAD18-*jspA*_E148A_*-HA* | This study |
| pJC720 | pDSW204-*jspA-HA* | This study |
| pJC730 | pDSW204-*jspA-HA-*_RBS_*-lppA-HA* | This study |
| pJC731 | pDSW204-*jspA-HA-lppA-HA* | This study |
| pJC733 | pDSW204-*jspA*_E148A_*-HA* | This study |
| pJC734 | pDSW204-*lppA-HA-jspA-HA* | This study |
| pJC735 | pDSW204-*jspA*_E148A_*-HA-*_RBS_*-lppA-HA* | This study |
| pJC736 | pDSW204-*jspA*_E148A_*-HA-lppA-HA* | This study |
| pJC737 | pDSW204-*lppA-HA-jspA*_E148A_*-HA* | This study |
|  |  |  |

^a^ Plasmid sequence files (in GenBank format and available as S1 File) contain construction details. Plasmids pJC608 and pJC609 were generated when missense mutations were introduced during PCR amplification of *lppA* to construct pJC606. Mutant alleles of *jspA* and *lppA* were generated using megaprimer PCR (Tyagi *et al.*, 2004).

**References**

Barnett, M.J., Oke, V., and Long, S.R. (2000) New genetic tools for use in the *Rhizobiaceae* and other bacteria. *Biotechniques* **29**: 240-242, 244-245.

Blondelet-Rouault, M.H., Weiser, J., Lebrihi, A., Branny, P., and Pernodet, J.L. (1997) Antibiotic resistance gene cassettes derived from the omega interposon for use in *E. coli* and *Streptomyces*. *Gene* **190**: 315-317.

Chen, E.J., Fisher, R.F., Perovich, V.M., Sabio, E.A., and Long, S.R. (2009) Identification of direct transcriptional target genes of ExoS/ChvI two-component signaling in *Sinorhizobium meliloti*. *J Bacteriol* **191**: 6833-6842.

Fields, A.T., Navarrete, C.S., Zare, A.Z., Huang, Z., Mostafavi, M., Lewis, J.C., Rezaeihaghighi, Y., Brezler, B.J., Ray, S., Rizzacasa, A.L., Barnett, M.J., Long, S.R., Chen, E.J., and Chen, J.C. (2012) The conserved polarity factor *podJ1* impacts multiple cell envelope-associated functions in *Sinorhizobium meliloti*. *Mol Microbiol* **84**: 892-920.

Gibson, K.E., Barnett, M.J., Toman, C.J., Long, S.R., and Walker, G.C. (2007) The symbiosis regulator CbrA modulates a complex regulatory network affecting the flagellar apparatus and cell envelope proteins. *J Bacteriol* **189**: 3591-3602.

Guzman, L.M., Belin, D., Carson, M.J., and Beckwith, J. (1995) Tight regulation, modulation, and high-level expression by vectors containing the arabinose PBAD promoter. *J Bacteriol* **177**: 4121-4130.

Khan, S.R., Gaines, J., Roop, R.M., 2nd, and Farrand, S.K. (2008) Broad-host-range expression vectors with tightly regulated promoters and their use to examine the influence of TraR and TraM expression on Ti plasmid quorum sensing. *Appl Environ Microbiol* **74**: 5053-5062.

Lee, T.S., Krupa, R.A., Zhang, F., Hajimorad, M., Holtz, W.J., Prasad, N., Lee, S.K., and Keasling, J.D. (2011) BglBrick vectors and datasheets: A synthetic biology platform for gene expression. *J Biol Eng* **5**: 12.

Marx, C.J., and Lidstrom, M.E. (2001) Development of improved versatile broad-host-range vectors for use in methylotrophs and other Gram-negative bacteria. *Microbiology* **147**: 2065-2075.

Mostafavi, M., Lewis, J.C., Saini, T., Bustamante, J.A., Gao, I.T., Tran, T.T., King, S.N., Huang, Z., and Chen, J.C. (2014) Analysis of a taurine-dependent promoter in *Sinorhizobium meliloti* that offers tight modulation of gene expression. *BMC Microbiol* **14**: 295.

Oke, V., and Long, S.R. (1999) Bacterial genes induced within the nodule during the Rhizobium-legume symbiosis. *Mol Microbiol* **32**: 837-849.

Quandt, J., and Hynes, M.F. (1993) Versatile suicide vectors which allow direct selection for gene replacement in gram-negative bacteria. *Gene* **127**: 15-21.

Tyagi, R., Lai, R., and Duggleby, R.G. (2004) A new approach to 'megaprimer' polymerase chain reaction mutagenesis without an intermediate gel purification step. *BMC Biotechnol* **4**: 2.

Weiss, D.S., Chen, J.C., Ghigo, J.M., Boyd, D., and Beckwith, J. (1999) Localization of FtsI (PBP3) to the septal ring requires its membrane anchor, the Z ring, FtsA, FtsQ, and FtsL. *J Bacteriol* **181**: 508-520.
