# Supplementary material for: A protease and a lipoprotein jointly modulate the conserved ExoR-ExoS-ChvI signaling pathway critical in Sinorhizobium meliloti for symbiosis with legume hosts
Source: PLoS Genet. 2023 Oct 23;19(10):e1010776. doi: 10.1371/journal.pgen.1010776 (PMC10659215; doi:10.1371/journal.pgen.1010776)
Supplement: Attachment — Submitted filename: JspA R1 response to reviewers 230909.docx [file pgen.1010776.s021.docx]

September 9, 2023

Aimee Shen

Academic Editor

PLOS Genetics

Lotte Søgaard-Andersen

Section Editor

PLOS Genetics

Dear Drs. Shen and Søgaard-Andersen:

We appreciate the detailed comments from the editors and reviewers regarding our previous submission (PGENETICS-D-23-00529); their efforts helped us to significantly improve our manuscript. We addressed all the comments in the decision letter regarding the manuscript. Please see our responses below. The original comments are in grey, while the responses are in black. Where applicable, we refer to line numbers in the revised manuscript.

The Reviewers recognized the importance of the authors’ findings in delineating a novel mechanism for controlling the activation of a conserved two-component signaling pathway in the Alphaproteobacteria that regulates interactions with eukaryotic hosts. They were particularly enthusiastic regarding the authors’ work in contributing to a broader understanding of the role that lipoproteins play in signaling. However, they noted a number of areas where the manuscript could be improved to merit publication. Please address their comments with particular attention to streamlining the presentation of the data and Introduction, refining their model regarding the role of LppA and JspA in signaling through the ExoR-ChvI-ChvG pathway (Reviewer 2), and testing the membrane localization of LppA and JspA directly. The rigor of the data presented would be improved by the addition of load controls to Figures 9 and 10, and Figure 2 is missing statistical analyses. Please also standardize the data presentation to show dots-on-plots.

We have shortened the Introduction and streamlined the data presentation. The abridged Introduction now contains only background information most relevant for understanding the study. Select images of calcofluor plates were moved into the supporting information section or eliminated, and measurements of calcofluor fluorescence are compiled in a new S2 Table. We combined the original Figs 9 and 10 into a single figure (now Fig 11) and consolidated the immunoblots assessing expression in *E. coli* into a single supplemental figure (S7 Fig). We also replaced Tables 1 – 4 with figures (Figs 5, 6B, and 6D) that facilitate visualization of the GUS assay data.

A model that depicts the role of LppA and JspA is now shown as Fig 1 (adapted from Fig 6B in the original manuscript). Cell envelope stress and acid stress have been added as potential signals for activating this aspect of the ExoR-ExoS-ChvI pathway.

We appreciate that the editors and reviewers share our curiosity regarding the mechanistic details of the signaling pathway. However, biochemical characterization of LppA and JspA is beyond the scope of the current study, which focuses on genetic interactions among LppA, JspA, and the ExoR-ExoS-ChvI pathway, and aligns with the Journal’s emphasis on genetics and genomics. We envision a future investigation that assesses the localization of LppA and JspA in the cell envelope, elucidates possible changes in response to stress, and provides further insights into how the pathway is activated.

We believe that the immunoblots showing changes in the steady-state levels of mature ExoR-FLAG (in the new Figs 11, S6, and S7) have sufficient loading controls. In contrast to the ExoR-V5 blot (in the new Fig 10B), which has no additional bands for comparison without the presence of the ATP synthase beta subunit, the ExoR-FLAG blots have other protein bands for comparison. Both JspA-HA and the precursor form of ExoR-FLAG show relatively uniform levels of expression across strains in these blots and serve as internal loading controls. We have labeled the pre-processed form of ExoR-FLAG on these blots to emphasize comparison between the precursor and mature forms. In addition, unlike other model systems, *S. meliloti* lacks well-characterized proteins that can serve as loading controls for Western blots. That is why we used commercial monoclonal antibodies against the *E. coli* protein to detect the beta subunit of the highly conserved ATP synthase. The beta subunit of ATP synthase has similar molecular mass as JspA-HA and interferes with visualization of that protein. Finally, we believe that the rigor and consistency of our analyses are supported by demonstrating similar changes in ExoR-FLAG levels across three different species (*S. meliloti*, *C. crescentus*, and *E. coli*).

In terms of statistical analyses and data presentation, we have changed all the plots to show data points as dots and included statistical significance where appropriate. For example, we show statistical significance for comparisons of calcofluor fluorescence (see the new Fig 3 and S2 Table).

Additional responses to specific comments are shown below.

Reviewer #1: Summary:

The manuscript from Bustamante et al. reports analysis of two presumptive lipoproteins LppA and JspA in Sinorhizobium meliloti (Sm) identified based on suppression of cell envelope defects in a Sm podJ mutant. These suppressor mutations also lead to decreased production of the EPS-1 polysaccharide. The JspA sequence identifies it as a periplasmic metalloprotease and the lipoprotein LppA is found to be required for its activity. Starting with the EPS phenotype, extensive genetic and expression analyses reveal that JspA and LppA act through the ExoR-ExoS-ChvI pathway that regulates EPS-1 synthesis in Sm and other rhizobia. Mutants manifest a competitive defect relative to wild type in nodulation assays on two different legumes. Mutants in jspA lead to elevated steady state ExoR levels, and overexpression leads to lower levels, and the presence of LppA is required for ExoR destabilization. Expression of these Sm proteins in Caulobacter crescentus and E. coli leads to a largely similar effect on ExoR turnover.

Major Comments:

This is a well-performed study that does a nice job of defining the impact of LppA and JspA and the way they act through ExoR-ExoS-ChvI. The conservation of these proteins suggests that they may have similar roles in other Alphaproteobacteria. The strength of the effects observed is surprisingly modest but statistically significant and consistent at almost all levels in the different analyses utilized. The role of periplasmic proteolysis is particularly relevant for ExoR- dependent regulation as low pH stimulates cleavage of ExoR, leading to high levels ExoS-ChvI activity. The paper is written somewhat opaquely about the relationship of the JspA-LppA system with the protease responsible for the low pH activation of ExoS-ChvI through ExoR cleavage. JspA does not appear to be responsible for the observed regulatory cleavage of ExoR, but it does seem to control the overall levels of the protein in the periplasm, which clearly can impact the regulatory patterns observed. It seems plausible that JspA and LppA play more general roles in the periplasm, and that ExoR is one of their substrates. The authors seem to lean heavily towards a specific role with the ExoR pathway, but remain somewhat obtuse about the low pH control. The authors should be more direct in their interpretation of their findings in the context of the ExoR proteolysis model.

We appreciate the careful reading of our manuscript. As we mentioned in the Introduction, while acidic pH appears to be a major signal for activating the ExoR-ExoS-ChvI pathway in related rhizobia, its role in *S. meliloti* remains ambiguous. Results from our study and others do not conclusively pin it as an environmental cue. We favor a model that explains the data most succinctly, that cell envelope stress (including exposure to acidic pH) can lead to degradation of ExoR and thus activation of the signaling pathway via JspA and LppA. To integrate this with all types of stress, more analyses will be required in future studies. We have revised the manuscript to emphasize that model in the new Fig 1. As before, we have included other possibilities and indicated the need for further study in the Discussion.

The manuscript presents an impressive amount of data and a wide range of experiments, but in some cases this seems to be inefficiently organized and presented. For example, the Calcofluor fluorescence plates could be presented as fluorescence values and the dilution plates could be supplemental information – do we really need to see all of these dilution plates? The Introduction also seems unnecessarily comprehensive.

As suggested by reviewer #1, we have shortened the Introduction, relying more on references to published reviews. We have also reduced the number of dilution plates shown, by modifying the old Fig 2 and 4 into the new Fig 3 and 6.

Minor Comments:

1.	Title – Both LppA and JspA are lipoproteins so this title is inaccurate.

We agree that LppA and JspA are both lipoproteins but recognize that a single protein may have multiple descriptors and wish to emphasize the proteolytic activity of JspA in the title. Unfortunately, the term "lipoprotease" has not been used to describe a lipoprotein with proteolytic activity. To avoid confusion in this regard, we state that both LppA and JspA are lipoproteins in the Results (lines 156 - 158) and Discussion (lines 514 - 515) and have revised the manuscript to include this information in the Abstract (line 35) and Introduction (lines 134 - 135) as well.

2.	L71. “Essential” is a loaded term – I would use something else – “critical” perhaps?

The adjective has been changed from “essential” to “required” (line 69): “bacterial production of required signaling molecules”.

3.	L230-1. Based on the sequences presented, it is predicted that lppA and jspA would localize to the outer membrane. Is there a reason to be so circumspect about this prediction? Although the role of lppA lipidation is genetically examined, that of jspA is not. Why not?

Sequence-based prediction of lipoprotein localization depends on limited data derived primarily from work done in *E. coli*, and the rules do not necessarily extend beyond *Enterobacteriaceae* [for example, see Zückert, 2014, *Biochim Biophys Acta.* 1843(8): 1509]. Detailed biochemical analysis, including identification of appropriate proteins for comparison, will be needed to demonstrate whether LppA and JspA are associated primarily with the inner or outer membrane in *S. meliloti*. The lipobox motif of JspA was previously shown to be critical for function by Arnold *et al*. (2017), discussed in lines 315 - 317, and thus was not evaluated in the current investigation, which focused on JspA’s proteolytic activity and its effect on steady-state levels of ExoR.

4.	L247. It is debatable whether the Ptau-jspA expressed in the jspA mutant causes a decrease in colony formation at 5 mM taurine, although this is clear at 10 mM. In stating that this effect is not observed in a lppA mutant, only 5 mM taurine is tested and 10 mM is not shown. Why is this not included to make the comparison valid? In contrast, the comparison to wt with Ptau-jspA is convincing.

Previously, we did not induce P_tau_-*jspA* expression in the *lppA* mutant with 10 mM taurine because we did not see an increase in calcofluor fluorescence with 5 mM taurine. We have now performed the analysis with 10 mM taurine and included the result in the new Fig 3. Inducing P_tau_-*jspA* expression in the *lppA* mutant with 10 mM taurine causes an increase in EPS-I production but does not lead to growth inhibition similar to that seen in the *jspA* mutant or wild type. Overexpression of *jspA* appears to overcome some of the requirement for LppA, but the results still indicate that LppA facilitates JspA function, as discussed in lines 185 - 188.

5.	L294. Most of the Miller unit values provided in the Table are strikingly low in the range around 10 units. What are the limits of detection with the assays as performed? Is there a chance that the increases are greater than represented and perhaps the decreases also underrepresented.

In our hands, the detection limit of GUS assays is below 0.1 unit. To measure low levels of enzymatic activity, we incubate the reactions overnight. If there is no *uidA* in the strain, the final absorbance at 415 nm would be the same as the blank, in which no cells were added. Our readings have generally been consistent over the years using the same instrument. One explanation for the low levels of activity is that the fusions are genomic and single-copy as opposed to plasmid-borne.

6.	L304. If the plasmid-borne gene is induced with taurine why would it be described as constitutive?

The wording has been changed to the following in lines 235 - 236: “assess the effects of *jspA* or *lppA* overexpression from a plasmid-borne, taurine-inducible promoter”.

7.	L361. Should indicate this is C. crescentus here to avoid confusion.

“*C. crescentus*” has been inserted in front of NA1000 in all places where it was missing (lines 297, 482, 988, and 1046).

8.	L501. Not sure I agree this is modest. Were growth rates calculated? Is it interesting that the impact of the catalytic site mutant is different between the two growth formats?

The adverb “modestly” was removed from the sentence (line 437) due to its subjective nature. The growth inhibition seemed more modest when viewed over an 18-hour period (see the new Fig S5A). We did not calculate growth rates previously, instead deeming visual representation of growth curves sufficient and also more accessible to readers. However, based on the reviewer’s suggestion, we have calculated growth rates and included the results in the new S9 Table, which contains data for all growth curves. Differences in growth between 48-well plates and flasks may be due to a number of factors, including variations in aeration or trace contaminants between culture vessels, as indicated in lines 437 - 440. To maintain the focus of the study, we have limited discussion on that topic.

9.	L522-3. Surprising that the authors do not mention that this increase seems specific to the processed form, and less so or not at all with the pre-processed form, consistent with the periplasmic location for this degradation activity.

We appreciate this advice and have inserted a sentence stating that steady-state levels of the ExoR-FLAG precursor form remains relatively uniform (lines 460 - 462).

10.	L531-3. Figure 9. One might argue that the lppA mutant is diminished for ExoR-FLAG in the strain with JspHA and chromosomal JspA (lane B5) compared to the strain with no JspA (lane B4) or even just the chromosomal copy (lane B6). Maybe there is ExoR degradation in the absence of LppA if sufficient JspA is expressed? Is it worth quantitating these ExoR bands?

Based on the results seen in Fig 3C, overexpression of JspA may be able to bypass some of the requirement for LppA. However, the effect appears too subtle for quantitation using Western blots, particular after induction for hours instead of days. For example, GUS assay results indicate that overexpression of JspA does not significantly increase *exoY* expression in the Δ*lppA* background (S5B Table, strain JOE4277; S5C Table, strain JOE4760).

11.	L539. IPTG-inducible Plac promoter

“IPTG-inducible” was inserted into the sentence (line 482).

12.	L547-8. It’s confusing to have both the untagged JspA and the Jsp-HA indicated in the same line for the gel key in 10A. I suggest adding a separate line for the two different expression constructs. The distinction between the black and red symbols is not particularly clear (especially for people, like this reviewer, with varying degrees of colorblindness).

We appreciate the advice and have changed the symbols for wild-type and mutant JspA-HA to # and ^, respectively, for all blots, to improve readability and accessibility.

13.	L552. It is also striking that the pre-processed form of ExoR is so much stronger in DH10B. Is this likely due to less efficient recognition of the ExoR signal sequence? This should at least be acknowledged in the results, even if no experiments were performed to determine the reason for it.

The steady-state levels of precursor ExoR-FLAG indeed appear relatively high in DH10B, possibly due to inefficient export. We have noted this and added that inefficient export is a possibility to the new S7 Fig legend (lines 1121 - 1123).

14.	L548-51. The 20 kDa species mentioned here should be marked or labeled in some way on 10A and S5A.

We have added this label to each of the relevant blot figures (new Figs 11, S6, and S7), with "deg" used to indicate this 20-kDa degradation product (lines 998 - 1000, 1088 - 1090, and 1115 - 1116). We also mention this prominent degradation product in the text (lines 458 - 460, 492-496).

15.	L595. In the case of Williams et al. this inhibition appeared to be ExoR-independent, in contrast to the LppA/JspA affect reported here.

The sentence was removed from the Discussion because it is speculative.

16.	L693. C. crescentus? Only this single species, correct, rather than multiple representatives of the genus as with Sinorhizobium.

Only the genus names were listed previously for stylistic consistency. “*Caulobacter*” has now been replaced with “*C. crescentus*”.

17.	L772. The sentence is confusing – is it that white nodules eventually turn pink or that at first they turn pink and then lose this pigmentation.

The sentence (lines 697 - 698) has been changed to indicate that nodules are initially white and then turn pink.

18.	L995. Presence OR absence. Also, is it clear that the leaky expression of chvI is depleted relative to the wild type?

The typo was corrected (line 930). We do not have a clear way to determine the extent of leaky expression, but the growth assays shown in S4 Fig suggest that ChvI expression is at least reduced, such that growth is inhibited in rich medium.

19.	L1049-50. Wild-type (+) or the jspAE148A mutant.

The suggestion was implemented (lines 985 - 986).

20.	L1061. I assume this was probed with a mixture of anti-FLAG and anti-HA antibodies as this appears to be a single blot and exposure, rather than two separate probings. This is in contrast to 10B in that respect.

The blots were all probed twice, and their images captured after each probing. For the *C. crescentus* NA1000 blot, there were sufficient anti-FLAG signals after the second probing with anti-HA to reveal all the relevant bands. The blots were not stripped between treatment with different antibodies, just washed. This is described in the Materials and Methods section (lines 763 - 767) and S6 Fig legend (lines 1100 -1102).

21.	Tables 1-3. These tables are awkwardly formatted in a way that makes comparisons cumbersome – there is too much text to navigate through and they take up too much space. For example, Table 1 lists out the same mutant designations three times – why not list these 1 time and array the data from the three different gene horizontally?

We have plotted representative GUS assay data (Fig 5) and formatted the tables as supporting information (S5 Table).

22.	Figure 10B – the asterisk for the band ascribed to the readthrough product is not mentioned in the results and is rather buried at the end of a long figure legend.

We have changed this marking to “read-through” in the new S7 Fig. This read-through product is also described in the Materials and Methods (lines 649 - 651) but does not seem to warrant discussion in the main text, as it is expressed at low levels compared to JspA-HA and LppA-HA individually.

Reviewer #2: In this paper, Bustamente and colleagues report that two novel lipoproteins, LppA and JspA, are regulators of the key two-component signaling pathway ExoS-ChvI in Sinorhizobium meliloti. The authors use deletion mutants and genetic analysis to determine that LppA and JspA work in the same pathway to positively regulate exopolysaccharide (EPS) production. These factors are likely to be sensors of stressors/signals and appear to trigger response by promoting the degradation of ExoR, a negative regulator of the system. Ultimately, these proteins promote EPS production and regulate other factors required for infection through the ExoR-ExoS-ChvI system. The importance of these proteins for host infection is shown by the competitive defect of lppA and jspA single mutants relative to WT strains, although no clear defect is present in monoculture infections of the mutants.

Overall, the study is well-conducted, and sufficient controls are included to support the authors conclusions. The discovery of two novel lipoproteins regulating the activation of a two-component system contributes to a growing understanding of the role that lipoproteins play in signaling across the Gram-negative envelope. The study could be strengthened by a few changes to the writing and experiments to confirm/support interpretations made by the authors, which are listed here below as major and minor points.

Major points

1.	The authors do not comment on where LppA and JspA are precisely localized (Lines 230-232). Ideally, confirming whether these proteins are localized to the IM or OM would provide more definitive idea as to the signals sensed by these proteins. This would also strengthen the comparisons the authors make to other lipoprotein envelope stress sensors, such as RcsF, which appear to be predominantly OM localized. Because the authors already have vectors expressing tagged versions of these proteins, conducting a localization experiment, such as sucrose density gradient fractionation, may be useful to include. Alternatively, it may be worth seeing if the introduction of a “canonical” Lol pathway avoidance signal to these proteins leads to different phenotypes, which may imply localization in the OM or IM normally, although this may be complicated by differences between species in Lol pathway function.

We greatly appreciate the reviewer’s suggestions; however, determining the subcellular locations of mature LppA and JspA is beyond the scope of the current study, which sheds light on genetic interactions among LppA, JspA, and the ExoR-ExoS-ChvI pathway, and is consistent with the Journal's focus on genetics and genomics. Rigorous assessment of JspA and LppA’s localization and its relevance to their activities is more appropriate for a future study. Moreover, any future study will need to include pioneering work to establish conditions and controls for membrane fractionation experiments, as these are not standard techniques in the *S. meliloti* model system, nor in related model bacteria. Regarding the alternative approach of introducing a “canonical” Lol pathway avoidance signal, indeed, the Lol pathway appears to function differently in alpha-proteobacteria. For example, a recent study in *C. crescentus* reported that LolB is not present in alpha-proteobacteria; instead, a bifunctional periplasmic LolA appears to compensate for LolB in lipoprotein insertion (Smith *et al*., 2023, *PNAS* 120: e2218473120). Complete characterization of the Lol pathway mechanisms in *S. meliloti* is not within the scope of our study discovering JspA and LppA.

2.	If IM-OM fractionation is not feasible, it would be useful to at least confirm that LppA and JspA are membrane-anchored in whole membrane preparations. This would strengthen the interpretation that C23S mutation in LppA impacts membrane anchoring. At this point, it’s difficult to make meaningful interpretations about the impact of LppA acylation because the C23S variant appears to be highly unstable, and its lack of change in calcofluor fluorescence could be attributable simply to low levels of protein present. It would be useful to create a periplasm-localized but stable version of LppA (e.g. by fusing mature LppA sequence to the signal peptide of a known periplasmic and/or IM-anchored protein). This would allow the authors to more definitively determine if the lipoprotein-nature/membrane anchoring of LppA is essential to its function versus the data provided by the unstable C23S mutant.

Again, we appreciate the reviewer’s insights and concrete suggestions, particularly regarding domain-swapping, but plan to defer this type of analysis for future investigation.

3.	Similarly, I think it would be important that the authors test whether mutating the putative N-terminal cysteine of JspA has similar impacts on JspA function as it does to LppA. If membrane-anchoring is important for LppA, it would also make sense that JspA must also be membrane anchored but this is not tested in this study.

Arnold *et al*. (2017) previously showed that introducing the C29S mutation into the lipobox motif of JspA reduced resistance against the NCR247 antimicrobial peptide, but the Q30D mutation did not alter resistance, suggesting that membrane anchoring, but not the exact location, is important for function. That study is referenced in the manuscript (please see lines 315 - 317). These mutations would be helpful in a future investigation that incorporates the reviewer’s suggestions for assessing the specific membrane location of JspA.

4.	Are there loading controls for the protein expression blots (e.g. Figure 9 and 10)? While the conclusions reached in the text appear to be broadly accurate, the presence of loading controls (e.g. presumably the inclusion of anti-ATPase B) would allow the authors to quantify protein expression levels and make more quantitative claims about the relative levels of ExoR present. Also, I’m not sure that the C. crescentus and E. coli heterologous expression experiments shown in Figure 10 contribute enough novel information to be included as a separate main text figure. I think that they could be combined into Figure 9 or included as a supplemental figure with no loss to the quality of the claims made in the text.

As discussed above (bottom of page 1), there are internal loading controls for the blots in the new Figs 11, S6, and S7. In particular, the relatively uniform levels of pre-processed ExoR-FLAG can be used for assessing changes in the steady-state levels of mature ExoR-FLAG.

Following the reviewer’s helpful advice, we have streamlined the presentation of ExoR-FLAG blots: we combined the original Figs 9 and 10A into the new Fig 11 and consolidated the blots for assessing heterologous expression in *E. coli* into a single supplemental figure (S7 Fig).

Minor points

1.	To better translate the authors’ mutant analysis to real impacts on infection, it would be useful test if the single amino acid substitutions (e.g. C23S in LppA and the enzymatically inactive E148A JspA) phenocopy their whole gene deletions in the plant infection model. Testing this could strongly support the in vivo mechanisms of these proteins in S. meliloti infection.

We appreciate the reviewer’s concern but believe that such analyses would be more fruitful when we have a better grasp of the activities of JspA and LppA and if we find that other domains of the proteins play additional roles.

2.	Lines 250-253: The way the data is presented is slightly confusing because the panels referred to in these lines are included in panels A and B, which were referred to earlier in the paragraph. It might be useful to take the bottom panels of A and B and combine them into a new panel that contains only the experiments where LppA or JspA are overexpressed in the opposing deletion mutant background.

We have revised the old Fig 2 into the new Fig 3 and simplified the presentation of the data.

3.	Lines 253-255: The connection between lack of growth inhibition seen when overexpressing JspA in an lppA mutant and overall conclusion (that “LppA and JspA act in concert …”) could use a bit more explanation.

We have edited the end of that paragraph to clarify that LppA and JspA require each other to accomplish their physiological activities (lines 185 - 188). At this point of data presentation in the manuscript, there is no clear explanation for the growth arrest yet.

4.	Line 320: The phrasing “LppA acts as a lipoprotein” might be somewhat confusing as lipoproteins are functionally diverse (as seen in the fact that JspA, a lipoprotein, is a protease). It might be useful to rephrase this to be more specific (e.g. “to determine if LppA membrane anchoring is important” etc.).

We have changed the phrase as follows: “To determine if membrane anchoring of LppA and proteolytic activity of JspA are critical for function” (lines 252 - 253).

5.	Line 341-342: While it’s very interesting that the mutants of LppA (G96W and A78S) possess phenotypes that differ from WT, I’m not sure if including these mutants contribute very much to the manuscript unless there is more work done as to their potential mechanism, especially considering that these mutants do not completely abrogate LspA function. It may be worth looking into the structure of LppA as predicted by AlphaFold (e.g. on the online AlphaFold database) to provide some rationale or hypothesis as to the potential effects of these mutations.

We have followed the reviewer’s insightful suggestion and included the predicted structure of LppA in S2 Fig. A brief description has also been added to the main text to suggest possible effects on tertiary structure (lines 276 - 279).

6.	Line 440-441: It would be clearer if this sentence was rephrased to refer to the loss of JspA and LppA instead of directly referring to the proteins themselves.

We have made the suggested change (lines 375 - 377).

7.	Because of the comprehensive nature of this paper, it would be nice if the authors include a final model figure which incorporates all of their conclusions from their data to tie everything together.

We agree that providing a model figure allows easier comprehension. Because the ExoR-ExoS-ChvI pathway is mentioned in the Introduction, we decided to include the model in the beginning of the manuscript as the new Fig 1. We also added possible inputs of cell envelope stress and acid stress to the figure.

Reviewer #3: I am extremely enthusiastic about this manuscript. It is an incredibly thorough genetic dissection of the the interaction with the predicted lipoprotein lppA and the predicted metalloprotease jspA with the exoR/exoS system and its regulation of the EPSI/flagella production switch. I would call it nearly flawless. On top of that, the manuscript does not stop with the genetics! It demonstrates that both JspA and LppA are required for degradation of ExoR, which is known to negatively regulate exoS/chvI sensor kinase system, which in turn, positively regulates expression of exoY.

The only change I recommend is a change in line 340 to state that 'these results are consistent with acylation at the Cys residue of the predicted lipobox allowing LppA...'

We thank the reviewer for the positive comments and have made the recommended change in the text (lines 272 - 274).

Thank you for your consideration. Please feel free to contact me if you have questions or concerns about the manuscript.

Sincerely,

Joseph C. Chen
