# Supplementary material for: A protease and a lipoprotein jointly modulate the conserved ExoR-ExoS-ChvI signaling pathway critical in Sinorhizobium meliloti for symbiosis with legume hosts
Source: PLoS Genet. 2023 Oct 23;19(10):e1010776. doi: 10.1371/journal.pgen.1010776 (PMC10659215; doi:10.1371/journal.pgen.1010776)
Supplement: Attachment — Submitted filename: JspA R2 response to reviewers.docx [file pgen.1010776.s022.docx]

October 6, 2023

Aimee Shen

Academic Editor

PLOS Genetics

Lotte Søgaard-Andersen

Section Editor

PLOS Genetics

Dear Drs. Shen and Søgaard-Andersen:

We appreciate the dedication that the editors and reviewers have shown for our manuscript (PGENETICS-D-23-00529R1). We have addressed all the comments in the decision letter regarding revision R1. Please see our responses below. The original comments are in grey, while the responses are in black. Where applicable, we refer to line numbers in the revised R2 manuscript (the final version without markup).

We thank the authors for their careful attention to the comments of the Reviewers, and I look forward to accepting this excellent manuscript after the changes to the terminology regarding strains and mutants is modified based on Reviewer 1's comments.

We have corrected the imprecise language used when referring to mutant strains throughout the manuscript. Please see the file that tracks changes between revisions R1 and R2.

Reviewer #1: Summary:

This extensively revised manuscript from Bustamante et al. describes studies on two presumptive lipoproteins LppA and JspA in Sinorhizobium meliloti (Sm) that they find impact the ExoR-ExoS-ChvI regulatory pathway through effects on ExoR stability in the periplasm.

Major Comments:

The authors have performed a commendable job of revising the manuscript in response to the initial review (particularly the comments of Reviewer 2 and my own comments). The manuscript includes a massive amount of data already, and I do agree with the authors in general that addition of extensive additional experimental data seems to go beyond reasonable expectations for a single paper. The study includes a wide range of experiments and approaches, and at least to me does a very impressive job of establishing the impact of lppA and jspA through the RSI pathway.

I did re-read the entire manuscript, and there was a minor issue that struck me on this re-reading that would be worth addressing. The authors use the term “strain” in a confusing way. WSM419 is a particular strain of S. medicae – a sub-lineage of the species. However, Sm1021 with an engineered deletion of jspA is most accurately described as a mutant of the strain 1021. The authors use these terms interchangeably, sometimes in the same sentence.

For example: - “While induction of lppA or jspA expression promoted EPS-I production in wild type or corresponding deletion strains, lppA expression in the ΔjspA mutant (Fig 3A, bottom row) and jspA expression in the ΔlppA mutant (Fig 3B, bottom row) failed to increase calcofluor fluorescence (S2B Table).” could clean up this terminology throughout the manuscript as it is confusing used.

Are the strains different from the mutants? Given that the manuscript DOES in fact include multiple strains as well as a wide variety of different mutants, the usage of this terminology should be standardized.

We appreciate the reviewer bringing this colloquialism to our attention: the term “deletion strains” was used as a shorthand for mutant strains carrying individual gene deletions. We have cleaned up the terminology regarding deletion mutants throughout the text. For the example provided by the reviewer, the phrase was changed to “While induction of *lppA* or *jspA* expression promoted EPS-I production in the wild type or corresponding deletion mutants” (lines 181 - 183).

In addition, in the Materials and methods, we have indicated that all *S. meliloti* strains used in the study are derived from Rm1021 and all *S. medicae* strains from WSM419 (lines 614 – 615). Each derivative is considered a distinct strain and assigned a number (Maloy and Hughes, 2007, *Methods Enzymol*. 421:3).

Minor Comments:

L82. Ref 22 has nothing to do with the statement. Perhaps the wrong reference? Brucella is not a rhizobial endosymbiont so perhaps just using a more broad descriptor would fix this problem?

We meant to indicate species that belong to the *Rhizobiales* group but recognize the error now. We have replaced the adjective “rhizobial” with “related”: “Mutations in ExoS and ChvI, as well as their orthologs in related endosymbionts, impair host colonization” (lines 81 – 82). This was the descriptor in the original manuscript but was altered during revision without noticing the implications.

In response to this comment, we have also corrected other instances when the terms “rhizobia” and “rhizobial” were improperly applied. Please see the file in which changes between revisions R1 and R2 have been highlighted.

Thank you for your consideration. Please feel free to contact me if you have questions or concerns about the manuscript.

Sincerely,

Joseph C. Chen
